# Increased genomic predictive ability in mango using GWAS-preselected variants and fixed-effect SNPs

**DOI:** 10.3389/fpls.2025.1664012

**Published:** 2025-10-29

**Authors:** Norman Munyengwa, Melanie J. Wilkinson, Daniel Ortiz-Barrientos, Natalie L. Dillon, Matthew Webb, Asjad Ali, Ian S. E. Bally, Alexander A. Myburg, Craig M. Hardner

**Affiliations:** ^1^ Queensland Alliance for Agriculture and Food Innovation, The University of Queensland, Brisbane, QLD, Australia; ^2^ School of the Environment, The University of Queensland, Brisbane, QLD, Australia; ^3^ Australian Research Council Centre of Excellence for Plant Success in Nature and Agriculture, The University of Queensland, Brisbane, QLD, Australia; ^4^ Australian Research Council Training Centre in Predictive Breeding for Agricultural Futures, The University of Queensland, Brisbane, QLD, Australia; ^5^ Queensland Department of Primary Industries, Mareeba, QLD, Australia; ^6^ Queensland Department of Primary Industries, Brisbane, QLD, Australia; ^7^ Department of Genetics, Stellenbosch University, Stellenbosch, South Africa

**Keywords:** genomic prediction, mango, GWAS-preselected variants, genome-wide association studies, whole-genome sequencing, prediction accuracy, population structure

## Abstract

Genomic selection (GS) using whole-genome sequencing (WGS) data has potential to improve breeding value accuracy in fruit trees, but previous studies have reported limited gains compared to high-density marker sets. Incorporating preselected variants identified through genome-wide association studies (GWAS) is a promising strategy to enhance the predictive power of WGS data. We investigated whether incorporating GWAS-preselected variants and fixed-effect markers into genomic best linear unbiased prediction (GBLUP) models improves predictive ability for fruit blush color (FBC), average fruit weight (AFW), fruit firmness (FF), and trunk circumference (TC) in mango (*Mangifera indica* L.). The study used 225 gene pool accessions from the Queensland Department of Primary Industries in Australia, with phenotypes collected between 1999 and 2024. Predictive ability was assessed using models that ignored or accounted for population structure using fixed principal components. Accounting for population structure led to substantial reduction in predictive ability across all traits, suggesting that initially high predictive abilities may have been partly driven by genetic differences between subpopulations. GWAS-preselected variants improved predictive abilities compared to using all WGS data, especially when population structure was accounted for in both parental and 5-fold cross-validation. Gains under parental validation reached 0.28 for AFW (from 0.30 to 0.58) and 0.06 for FBC (from 0.44 to 0.50). In 5-fold cross validation, gains were up to 0.16 for AFW (from 0.32 to 0.48) and 0.10 for FBC (from 0.35 to 0.45). This suggests that prioritizing markers that better capture relationships at causal loci can improve predictive ability. Fixed-effect SNPs improved predictive ability of WGS data, particularly for FBC, with increases of up to 0.18 (from 0.44 to 0.62). The combination of GWAS-preselected variants and fixed-effect markers yielded the highest improvements in predictive ability for FBC and TC. GWAS identified 5 trait-associated SNPs for FBC, 11 for AFW, and 8 for TC. These results demonstrate that leveraging GWAS-preselected variants and fixed-effect SNPs improves predictive ability, potentially enhancing breeding efficiency in fruit trees.

## 1 Introduction

Mango (*Mangifera indica* L.), the world’s fifth most produced fruit crop, holds major economic value due to its global consumption and diverse applications (Srivastav et al., 2023). While global production exceeds 50 million tons, Australia contributes less than 0.2%, with an estimated 61,474 tons produced annually, 89% of which is consumed domestically (Bally and De Faveri, 2021; Bally et al., 2021). Genetic improvement of mango is essential to enhance productivity and to meet evolving market demands. Key breeding goals include dwarf or semi-dwarf tree architecture suitable for high-density orchards (Mahmud et al., 2023; Reddy et al., 2003), attractive skin color, and market-specific fruit weight (Bally et al., 2009). Genetic gain in conventional mango breeding is primarily constrained by lengthy breeding cycles exceeding 20 years, with juvenility alone accounting for nearly half of this duration (Bally and Dillon, 2018). New breeding approaches that can reduce the breeding cycle length are greatly needed to accelerate genetic gains in mango breeding programs.

Genomic selection (GS) has great potential to shorten breeding cycles in horticultural fruit trees by predicting genetic values (breeding or clonal) of unphenotyped individuals at the juvenile stage using statistical models trained on a training set with both genotypic and phenotypic data (Meuwissen et al., 2001). Proof of concept studies in apple (Muranty et al., 2015; Roth et al., 2020), macadamia (O’Connor et al., 2021), and eucalyptus (Suontama et al., 2019) have demonstrated that GS can accelerate genetic gain per unit of time compared to conventional breeding by shortening the cycle length, primarily through skipping progeny testing. However, in oil palm, GS did not yield sufficient prediction accuracy for some key traits to justify skipping progeny testing (Cros et al., 2017), underscoring the importance of accurate genetic value prediction for effectively implementing GS in tree crops.

The genomic best linear unbiased prediction (GBLUP) model (VanRaden, 2008) is one of the most widely used approach for genomic prediction due to its flexibility and computational efficiency (Barreto et al., 2024). The GBLUP model estimates breeding values of selection candidates using a genomic relationship matrix (GRM), which aims to capture relationships among individuals at quantitative trait loci (QTLs). However, it assumes that all markers contribute equally to genetic variance (Meuwissen and Goddard, 2010), a limitation when a few major loci account for a substantial portion of trait variation. This can lead to underestimation of the contribution of major loci to genetic variation, and consequently, reduced genetic gain from GS (Bernardo, 2014). To address this, several studies have incorporated key trait-associated markers as fixed or random effects in GBLUP models, resulting in improved prediction accuracy (Bernardo, 2014; Chen et al., 2023; Hardner et al., 2022; Kostick et al., 2023).

Whole-genome sequencing (WGS) data has been proposed to improve the accuracy of genomic prediction by capturing QTL variants directly rather than relying on the linkage disequilibrium (LD) between markers and unobserved QTLs (Meuwissen et al., 2016). However, prior research has demonstrated that to enhance genomic prediction accuracy with WGS data, predictions should utilize preselected variants based on their association with target traits, such as those identified through genome-wide association studies (GWAS) (Liu et al., 2023; Raymond et al., 2018; Warburton et al., 2020; Wei et al., 2023; Ye et al., 2020). This is because not all markers in WGS data are causative or in strong LD with causative mutations for the target trait (van Binsbergen et al., 2015); instead, many may introduce noise into the prediction model, ultimately reducing prediction accuracy (Raymond et al., 2018). GWAS-preselected variants from WGS data may enhance prediction accuracy in GBLUP models by enabling the construction of trait-specific GRMs that prioritize causative mutations or markers in LD with them, thereby better capturing genetic relationships at causal loci. Although GWAS-preselected variants from WGS data have shown improved prediction accuracy in livestock (Jang et al., 2023a; Raymond et al., 2018; Veerkamp et al., 2016), this approach remains largely unexplored in fruit trees, including mango.

Genome-wide association studies (GWAS) remain the most widely used approach for identifying trait-associated single nucleotide polymorphisms (SNPs) and prioritizing markers for genomic prediction based on their potential causal effects. However, most studies employed single-locus GWAS (SL-GWAS) models, which test markers individually and have limited detection power for polygenic traits (Wang et al., 2016). The ability to detect causal variants is further influenced by factors such as effective population size (*Ne*), LD structure, GWAS sample size, and the statistical model used (Jang et al., 2023b). For instance, detection power is enhanced and sample size requirements are reduced for GWAS in populations with high *Ne* and low LD (Misztal et al., 2021), whereas small *Ne* increases long-range LD and noise, reducing detection power. To date, *Ne* has not been estimated in mango. In addition, most genomic prediction studies using GWAS-preselected variants have relied on a single GWAS methodology for variant discovery, limiting comparison across models. This represents a key research gap. To address this, we evaluate genomic prediction performance using GWAS-preselected variants identified from three multi-locus GWAS methods: Bayesian-information and Linkage-disequilibrium Iteratively Nested Keyway (BLINK) (Huang et al., 2019), the Fixed and random model Circulating Probability Unification (FarmCPU) (Liu et al., 2016) and the Multi-loci Mixed Linear Model (MLMM) (Segura et al., 2012). We also compare these with a single-locus approach, the general linear mixed model (GLMM).

A key challenge in genomic prediction is population structure, defined as the presence of genetically distinct subgroups with divergent allele frequencies (Jacquin et al., 2025). If unaccounted for, population structure can bias genomic estimated breeding values (GEBVs) and inflate estimates of selection accuracy (Riedelsheimer et al., 2013; Werner et al., 2020). Addressing population structure is especially critical in perennial tree crops, where training populations often represent broad genetic diversity to minimize phenotyping demands across populations or generations, given the long breeding cycles and extended juvenile phases (Brault et al., 2022). Despite its potential to confound predictions, population structure is frequently overlooked, especially when perceived to be weak. A common strategy used to account for population structure is to include principal components (PCs) derived from principal component analysis (PCA) of the GRM as fixed-effect covariates in prediction models (Hayatgheibi et al., 2024).

To the best of our knowledge, there are currently no published reports of genomic prediction in mango, and the use of GWAS-preselected variants from WGS data remains largely unexplored in tree crops. This represents a significant gap in the application of GS in mango and other fruit trees. To address this, we aimed to develop and evaluate strategies for improving genomic predictive ability for key traits in mango using WGS data. Specifically, we: (i) assessed the power of GWAS using multi-locus and single-locus models, (ii) evaluated the impact of increasing marker density to WGS level on predictive ability, (iii) evaluated whether predictive ability could be increased by using GWAS preselected variants, (iv) assessed the impact of incorporating significant GWAS loci as fixed effects in GBLUP models on predictive ability, and (v) investigated the impact of population structure on predictive ability. Together, these analyses inform strategies for optimizing genomic selection in mango.

## 2 Materials and methods

### 2.1 Germplasm and trial design

This study used 225 mango (*Mangifera indica* L.) accessions from the gene-pool collection of the Queensland Mango Breeding Program (QMBP), maintained by the Queensland Department of Primary Industries (DPI) in Australia. This collection comprises historical cultivars from 24 countries and progenies from advanced selections, capturing a broad spectrum of *Mangifera indica’s* genetic diversity (Wilkinson et al., 2025). The accessions exhibit strong population structure, divided into two primary sub-populations: 33 individuals of Southeast Asian origin and 192 of Indian ancestry (Wilkinson et al., 2022). Among the 225 gene-pool accessions, 41 are used as parents for the QMBP breeding population (**Supplementary Table 1**). None of these parental accessions originated from Southeast Asia. The trees were grown at the Walkamin Research Station (WRS) and assessments of fruit quality traits and tree growth were conducted from 1999 to 2024.

### 2.2 Phenotypic data

#### 2.2.1 Trunk circumference

Trunk circumference (TC), an indicator of tree vigor, was measured using a tape measure positioned 10 cm above the graft union. Due to differences in planting times, the trees were assessed at different ages, resulting in unbalanced data. We used TC data for trees assessed at the ages of 9 (TC9, 200 unique accessions) and 12 (TC12, 199 unique accessions) years (total of 207 unique accessions) due to the availability of a relatively large number of individuals assessed in these years.

#### 2.2.2 Fruit quality traits

Physiologically mature fruits were harvested from the outer tree canopy, where they were exposed to sunlight. The fruits were washed thoroughly with a detergent, treated with a fungicide dip (1.0 ml L-1 Fludioxonil (230g/L)) for five minutes at 52 °C to control anthracnose. They were then stored in a ripening room maintained at 22°C until they developed a soft texture. Fruit blush color (FBC) was assessed in 220 accessions over at least two seasons, using ten ripe fruits from each accession. FBC of the ripened fruit was rated on a categorical scale, in order from least to most desirable: no blush, orange, pink, pink-red, red, and burgundy. FBC categorical data was converted to a numerical scale as: no blush or yellow = 0, orange = 1, pink = 2, pink-red = 3, red = 4, and burgundy = 5.

The average fruit weight (AFW) in grams (g) was calculated across 222 accessions using the weight of ten fruits at the eating ripeness stage. Fruit firmness (FF) was measured in 221 mango accessions using an analogue firmness meter. Not all accessions were assessed for the three fruit quality traits in every season due to the irregular bearing of some cultivars and differences in planting seasons, resulting in unbalanced data.

### 2.3 Molecular data

Genomic DNA extraction, whole genome sequencing and variant calling followed the protocols outlined by Wilkinson et al. (2025), using the same set of 225 mango gene-pool accessions utilized in this study. Briefly, genomic DNA was extracted from young mango leaf tissues using the modified cetyltrimethylammonium bromide (CTAB) method. Whole genome sequencing (WGS) was performed on all 225 accessions, with the 41 parental accessions sequenced at 40X coverage and the remaining 184 individuals at a depth of 15X. Joint SNP calling was performed using GATK4 software (Poplin et al., 2018), and trimmed paired-end reads were aligned to the *M. indica* ‘Alphonso’ reference genome (Wang et al., 2020) to identify physical position. This resulted in a total of 44,125,383 SNPs.

To generate a high-quality SNP dataset, a series of quality filtering steps using VCFtools (Danecek et al., 2011) were applied. Data points with a read depth below five were set to missing, and SNPs exhibiting more than 20% missing data across the population were discarded. To ensure the inclusion of only the most reliable variants, we imposed a maximum mean read depth of 50, removed SNPs with a minor allele frequency (MAF) below 0.05, and applied a Hardy-Weinberg equilibrium *p*-value cut-off of 1e-6 to eliminate potential genotyping errors. Following these stringent quality control measures, 10,172,985 SNPs remained for downstream analyses. Missing markers in the final dataset were imputed using the Hidden Markov Model (HMM) implemented in Beagle 5.4 (Browning et al., 2018).

### 2.4 Estimation of effective population size ( $N_{e})$ and linkage disequilibrium

To assess genetic diversity within the QMBP gene-pool collection, we estimated recent historical *N_e_* for the 225 accessions based on LD between pairs of markers, as implemented in GONE software (Santiago et al., 2020). This method estimates the *N_e_* from the variance of progeny number, which is equal to the number of breeding individuals (*N*). To minimize downward bias in *N_e_* estimates due to elevated LD (Waples et al., 2016), we used 815,255 independent SNPs derived by pruning the initial set of ~10 million SNPs. Pruning was performed in PLINK 2.0 (Purcell et al., 2007) by removing one SNP from each pair with a squared correlation coefficient (*r*²) > 0.2 within a 35-SNP sliding window. Additionally, *N_e_* was estimated for each of the two sub-populations defined by Wilkinson et al. (2022), as population structure can bias *N_e_* estimates (Santiago et al., 2020). Furthermore, *N_e_* was estimated for the parental accessions in the QMBP to evaluate whether sufficient genetic diversity exists to sustain long-term genetic gains within the breeding program. Analyses were conducted using default GONE software parameters.

To evaluate LD decay with physical distance among the 225 gene-pool accessions, pairwise estimates of LD were calculated using the squared correlation of allele frequencies (*r²*) for all SNP pairs within 1 Mbp windows across the entire set of 10,172,985 SNPs. The distance at which *r²* decayed to 0.2, commonly regarded as the minimum threshold for high genomic prediction accuracy (Calus et al., 2008), was determined separately for each chromosome using PopLDdecay (Zhang et al., 2019).

### 2.5 GBLUP model implementation and parameter estimation

Linear mixed models were used to fit residual maximum likelihood (REML) as implemented in the R package ASReml-R 4 (Butler et al., 2023), within a GBLUP framework to estimate model parameters and predict random and fixed effects for all traits. When a GRM was ill-conditioned (i.e. not positive-definite), bending was applied to allow for matrix inversion, as implemented in the ASRgenomics R package (Nazarian and Gezan, 2016). The linear mixed model used to predict the genomic estimated breeding values (GEBVs) of mango individuals is given in Equation 1:


(1) y=Xb+Za+e

Where y was the vector of phenotypic measurements, X was the design matrix relating phenotypic records to the vector of fixed effects (the intercept for all traits, age of tree at assessment for trunk circumference, significant markers for models that included these as fixed effects, and the first six principal components for models that accounted for population structure) denoted by b , Z was the design matrix linking phenotypic records to the additive genomic effects of the mango accessions, a was the vector of additive genomic effects, and e represented the random residual effects. We assumed the following distributions for the four traits: $a\;\sim \;\text{N}\left(0,{\text{σ}}_{\text{a}}^{2}G\right)$ and $e\;\sim \;\text{N}\left(0,\;I{\text{σ}}_{\text{e}}^{2}\right)$ , where G was an n ×n symmetric and positive-definite additive GRM which described the additive genomic relationships among all pairs of individuals in both the training and validation sets. The additive genomic variance explained by the set of SNPs in each analysis was denoted by ${\text{σ}}_{\text{a}}^{2}$ . The residual variance was denoted by ${\text{σ}}_{\text{e}}^{2}$ , and I was an n ×n identity matrix. For trunk circumference, ${\text{σ}}_{\text{a}}^{2}\;$ was replaced by the additive genomic-by-age-at-assessment covariance matrix, $G_{A\times Age},$ and the 2 ×2 variance-covariance matrix of residual effects were modelled using a CORGH variance structure, assuming correlated heterogeneous variances among observations across the two ages of assessment (age 9 and 12). In this case, ${\text{σ}}_{\text{e}}^{2}$ was replaced with the residual variance-covariance matrix capturing both the heterogeneous residual variances and the residual correlation between ages. The additive genomic relationship matrix (**G**) for each marker set was estimated using the method described by Yang et al. (2010). Individual narrow-sense heritability ( ${\hat{h}}^{2}$ ) for each specific trait was estimated as ${\hat{h}}^{2}=\sigma_{a}^{2}/\left(\sigma_{a}^{2}+\sigma_{e}^{2}\right)$ . The Akaike Information Criteria (AIC) was used to assess the quality of model fit.

#### 2.5.1 Model validation

Two approaches were used to validate genomic prediction models in this study. In the first cross-validation approach (parental validation), own phenotypes of the 41 gene-pool accessions that are being used as parents in the QMBP served as an independent dataset for model validation, while the remaining gene-pool accessions served as the training population. Predictive ability was estimated as the Pearson correlation between the phenotypes predicted by the linear mixed models (GEBVs) and the observed phenotypes of parental accessions, $r\left(\hat{y,\;\;}y\right)$ .

To provide a more robust evaluation of model performance, a second validation approach involving random 5-fold cross-validation (5-fold CV) was also implemented. In this approach, the entire gene pool collection was randomly partitioned into five subsets in which each subset consisted of 20% of the accessions. For each fold, four subsets (80% of total individuals) were used for model training and the remaining fold (20% of the accessions) for model validation. Predictive ability was calculated as the Pearson correlation between the GEBVs and the observed phenotypes after each 5-fold CV run. To ensure stability and reliability of the predictive ability estimates, the 5-fold CV procedure was repeated five times. Thus, 25 correlation values were calculated for each model. For trunk circumference, only phenotypic data collected from trees aged 12 years were used for validation. The bias of predictions was calculated as the regression of phenotypes on GEBVs for individuals in the validation set.

### 2.6 Linkage disequilibrium pruning of WGS data

To evaluate whether increasing marker density to WGS level enhances genomic predictive ability, we performed GP using the full set of available WGS markers (~10 million SNPs) and lower-density marker sets (~2 million, ~800k, ~80k, ~20k, and ~10k SNPs). These reduced marker sets were generated by pruning correlated markers based on LD thresholds. The LD pruning thresholds were chosen arbitrarily to generate a range of marker densities. LD pruning was performed using PLINK 2.0 (Purcell et al., 2007) to remove one SNP from each pair if their squared correlation (*r²*) exceeds a user-defined threshold within a specified window. For example, the ~2 million SNP dataset (LD_2mil) was created by pruning one of each pair of SNPs if their *r^2^* value exceeded 0.2 within a window size of 15 SNPs, shifting the window 10 SNPs forward and repeating the procedure. More stringent LD thresholds were applied to derive lower-density marker sets, as detailed in **Table 1**. The final datasets - LD_2mil (~2 million SNPs), LD_800k (~800k SNPs), LD_80k (~80k SNPs), LD_20k (~20k SNPs), and LD_10k (~10k SNPs) were used to assess the impact of marker density on predictive ability.

**Table 1:** Description of marker sets including whole-genome sequencing (WGS) data and LD-pruned markers.

Scenario	*R* ^2^	Window size	Number of SNPs	*R* ^2^ between adjacent SNPs
WGS	NA	NA	10, 172, 985	0.33
LD_2mil	0.2	15	2,016,911	0.11
LD_800k	0.2	35	815,255	0.08
LD_80k	0.2	1,500	82,504	0.03
LD_20k	0.1	8,000	20,523	0.01
LD_10k	0.1	100,000	10,068	0.01

PLINK 2.0 was used to prune one of each pair of correlated SNPs at an arbitrarily chosen LD threshold using WGS data. For example, the LD_2mil scenario was created by pruning one of each pair of SNPs if their r^2^ value exceeded 0.2 within a window size of 15 SNPs, shifting the window 10 SNPs forward and repeating the procedure again.

### 2.7 Accounting for population structure

To evaluate the impact of population structure on predictive ability, the top six principal components (PCs) derived from principal component analysis (PCA) of the GRM were included as fixed effects in GBLUP models. Since LD can affect PCA analysis (Campoy et al., 2016), we conducted PCA using a GRM constructed using a set of ~80k (LD_80k) unlinked markers derived from LD pruning of the ~10 million WGS markers. We selected the top six PCs to represent population structure based on their relative contributions to global molecular variance. Individually, these PCs accounted for between 2.5% and 10% of the molecular variance, and together they explained 33% of the total variation in the mango gene pool collection. The predictive ability of models that included fixed PCs was compared to that for models that did not include this adjustment.

### 2.8 Genome-wide association study

We performed GWAS using the LD_2mil marker set to identify trait-associated markers and establish an association-based criterion for preselecting SNPs from WGS data for use in genomic prediction. Although GWAS for the same traits and phenotypic data was conducted in the original study by Wilkinson et al. (2025), our reanalysis aimed to enhance statistical power by leveraging a denser marker set and multi-locus GWAS methods. In this study, we evaluated three multi-locus GWAS methods: (1) the MLMM (Segura et al., 2012), (2) BLINK (Huang et al., 2019), and (3) FarmCPU (Liu et al., 2016). The MLMM employs a stepwise regression approach to iteratively incorporate the most influential markers (pseudo quantitative trait nucleotides: pseudo-QTNs) as covariates to account for population structure. The BLINK approach accounts for population structure using pseudo-QTNs selected using LD information and optimized for Bayesian information criterion (BIC), while FarmCPU employs the fixed-bin approach to select pseudo-QTNs, assuming a uniform distribution of pseudo-QTNs across the genome. All three multi-locus GWAS methods were implemented using GAPIT 3 (Wang and Zhang, 2021). For comparison, a single-locus GWAS was performed using a GLMM implemented in PLINK 2.0 (Purcell et al., 2007).

To account for population structure in GWAS analyses, both multi-locus and single-locus methods incorporated the first six PCs derived from PCA of the GRM as fixed effects as described above. A marker was considered significant if it surpassed the Bonferroni threshold (-log(p) = 7.61). For TC, GWAS was conducted separately for trees assessed at the ages of 9 and 12 years. To perform GWAS for variant preselection or the identification of fixed-effect SNPs, GWAS analyses were exclusively conducted using individuals from the training population. This exclusion was implemented to minimize the bias in GEBVs that could arise from discovering markers in the same population used for model validation.

### 2.9 Incorporation of GWAS results in GBLUP models

To evaluate whether predictive ability using WGS data can be improved by prioritizing markers based on potential LD with QTLs, we created marker subsets containing preselected variants identified using GWAS approaches described earlier. Markers were first ranked in descending order of estimated effect from GWAS (-log10(p-value)), with the most statistically significant SNPs selected first. Different densities of preselected variants were evaluated as top 1,000, 10,000, 15,000, 20,000, 30,000, 50,000, and 100,000 SNPs. Markers preselected through GWAS conducted using BLINK, FarmCPU, MLMM, and the GLMM methodologies are referred to as TOP-BLINK, TOP-FarmCPU, TOP-MLMM, and TOP-GLMM, respectively. Genomic predictive ability from GBLUP models using additive GRMs based on preselected variants from different GWAS models and unselected marker sets (WGS data and LD-pruned data) were compared.

To test the hypothesis that fitting significant SNPs from GWAS as fixed effects enhances predictive ability, the additive genetic effects of significant markers identified by at least two GWAS methods, hereafter referred to as reliable SNPs, were added to GBLUP models as fixed effects. These reliable SNPs were identified using GWAS in the training population. In models incorporating fixed-effect SNPs, reliable SNPs were excluded from GRM construction, and their best linear unbiased estimates (BLUEs) were added to the GEBVs prior to model validation. The fixed-effect SNPs were added to models based on GWAS-preselected variants, WGS data, and LD-pruned marker sets.

## 3 Results

### 3.1 Effective population size and linkage disequilibrium

The effective population size (*N_e_*) varied considerably between sub-populations within the QMBP’s mango gene-pool collection. The overall *N_e_* for the entire gene-pool collection was estimated to be 113. Subpopulation-specific estimates revealed relatively high *N_e_* values for non-Southeast Asian accessions (*N_e_* = 129) and for individuals currently used as parents in the QMBP (*N_e_* = 104). In contrast, the Southeast Asian accessions exhibited a markedly lower effective population size (*N_e_* = 29).

**Figure 1:**
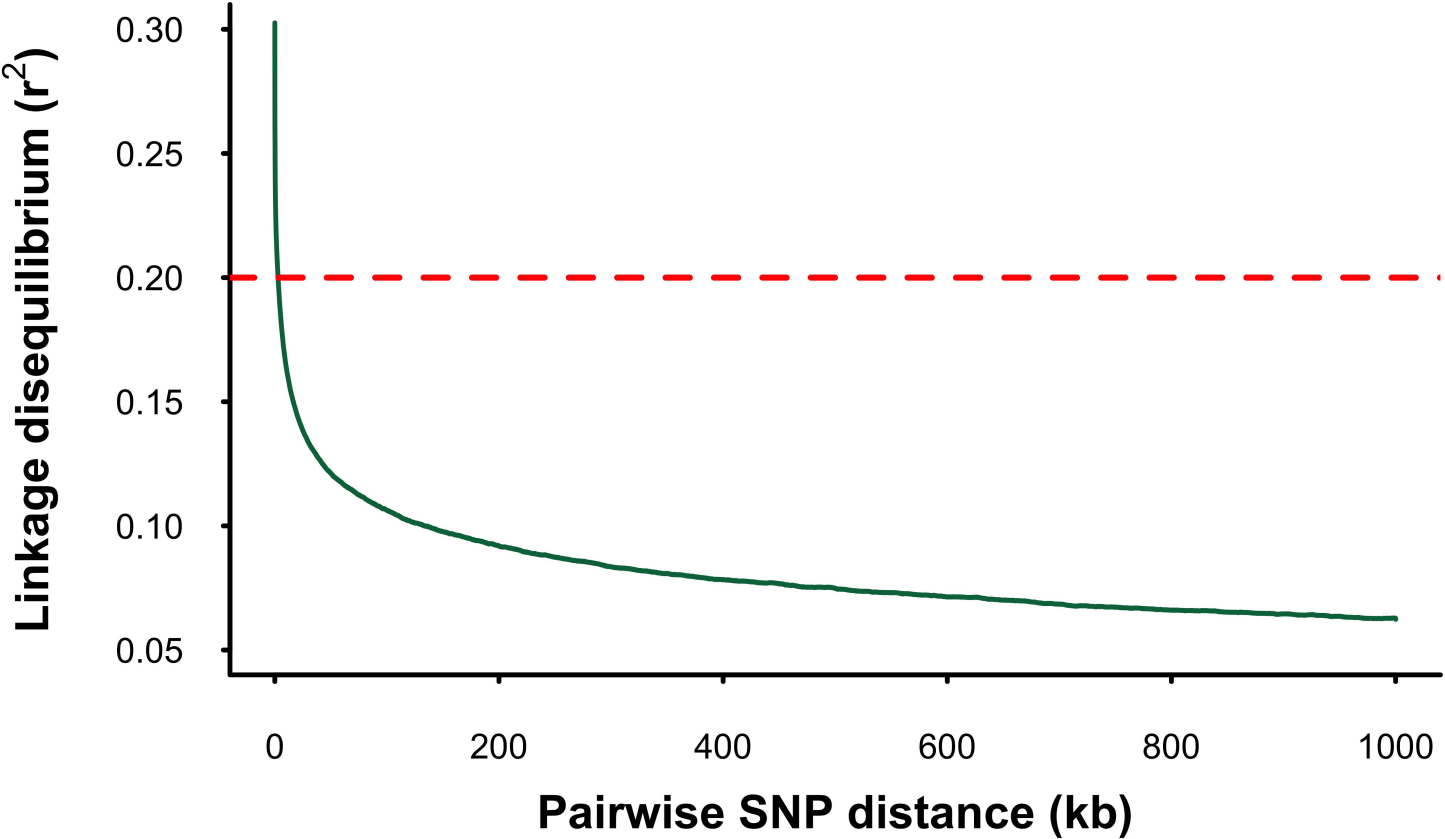
Linkage disequilibrium (LD) decay in the mango gene pool. The X-axis shows the physical distance between SNPs in kilobases (kb), and the Y-axis represents the squared correlation (r²) between allele frequencies. The dotted line marks the threshold of r² = 0.2.

Linkage disequilibrium (LD) decayed sharply with increasing physical distance between markers. The *r^2^* estimates between pairs of SNPs dropped below the widely accepted critical threshold for accurate genomic prediction (*r* ^2^ = 0.20) within 3.6 kb (**Figure 1**). The mean genome-wide *r^2^* between adjacent SNPs across all chromosomes in WGS dataset was 0.33. In contrast, the mean *r^2^* values for the LD-pruned marker subsets (LD_2mil, LD_800k, LD_80k, LD_20k, and LD_20k, and LD_10k) were substantially lower (**Table 1**).

### 3.2 Phenotypic analysis

We observed substantial to relatively low phenotypic variation across the evaluated traits in the mango gene pool (**Supplementary Table 2**). The greatest variability was observed for FBC and AFW, with coefficients of variation (CV) of 88.5% and 42.4%, respectively, indicating pronounced differences in pigmentation and fruit weight among accessions. In contrast, FF showed moderate variability (CV = 28.3%), while TC at ages 9 and 12 showed relatively lower variation (CV = 21.1% and 19.3%, respectively), with mean values of 50.4 cm and 56.06 cm. The density distributions of TC (**Supplementary Figure 1**) reveal a rightward shift from age 9 to 12, reflecting overall tree growth.

### 3.3 Heritability

Estimates of narrow-sense heritability ( ${\hat{h}}^{2}$ ) based on the full marker set (WGS data) varied widely across traits and models, revealing notably high heritabilities for FBC ( ${\hat{h}}^{2}$ =0.98) and AFW ( ${\hat{h}}^{2}$ = 0.95), but considerably lower estimates for FF ( ${\hat{h}}^{2}$ =0.26) and TC ( ${\hat{h}}^{2}$ =0.33) (**Supplementary Table 3**). Marker density exerted minimal overall impact on heritability estimates; however, contrary to expectations, an increase in marker density from ~10k (LD_10k) to full WGS coverage led to a reduction in heritability estimates for TC from 0.40 to 0.33. Incorporation of the first six principal components derived from the GRM as fixed effects intended to control for population structure resulted in only subtle changes in ${\hat{h}}^{2}$ across all traits (**Supplementary Table 4**).

Optimal model fits as indicated by lower AIC values were generally observed with intermediate to high marker densities, suggesting that an optimal balance exists between capturing genetic variation and avoiding over-parameterization. Moreover, prediction models employing GRMs constructed from GWAS-preselected variants consistently had better model fit than models based on the full WGS dataset.

### 3.4 Genomic prediction using WGS data

#### 3.4.1 Predictive ability with WGS data and effect of marker density on predictive ability

Genomic predictive ability varied across traits, marker density, and validation strategy (**Table 2**, **Supplementary Table 7**), and generally aligned with the narrow-sense heritability estimates ( ${\hat{h}}^{2}$ ). When considering predictions based on WGS data and baseline GBLUP models (i.e., models without population structure correction or fixed-effect SNPs), higher predictive abilities (PA) were observed for highly heritable traits and lower predictive abilities for traits with lower ${\hat{h}}^{2}$ . Under the parental validation strategy, the highest predictive abilities were observed for FBC and AFW (PA = 0.67 for both traits), followed by TC (PA = 0.54), with FF showing the lowest predictive ability (PA = 0.41). A similar trend was observed in the 5-fold cross-validation (CV) strategy, where predictive abilities for AFW (0.65) and TC (0.57) were comparable to those from the parental validation (**Supplementary Table 7**). However, the predictive ability for FBC increased substantially under the 5-fold CV strategy (0.80), while that for FF decreased markedly (0.28), relative to the parental validation results.

**Table 2:** Genomic predictive abilities for fruit blush color (FBC), average fruit weight (AFW), fruit firmness (FF) and trunk circumference (TC) across different marker sets and prediction models under parental validation.

Trait	Scenario	Marker set									
		LD_10k	LD_20k	LD_80k	LD_800k	LD_2mil	WGS	TOP-BLINK	TOP_FarmCPU	TOP-MLMM	TOP-GLM
FBC	GBLUP	0.60	0.65	0.65	0.66	0.66	0.67	0.70	0.71	0.67	0.66
	GBLUP + fixed PCs	–	–	–	–	–	0.44	0.45	0.50	0.37	0.33
	GBLUP + fixed-SNPs	–	–	–	–	–	0.74	0.77	0.74	0.68	0.70
	GBLUP + fixed-SNPs + fixed PCs	–	–	–	–	–	0.62	0.69	0.64	0.51	0.48
AFW	GBLUP	0.59	0.65	0.66	0.67	0.67	0.67	0.78	0.68	0.70	0.68
	GBLUP + fixed PCs	–	–	–	–	–	0.30	0.37	0.58	0.48	0.54
	GBLUP + fixed-SNPs	–	–	–	–	–	0.68	0.78	0.69	0.71	0.69
	GBLUP + fixed-SNPs + fixed PCs	–	–	–	–	–	0.30	0.36	0.59	0.48	0.55
FF	GBLUP	0.35	0.38	0.40	0.41	0.41	0.41	0.43	0.43	0.40	0.45
	GBLUP + fixed PCs	–	–	–	–	–	0.30	0.34	0.34	0.27	0.35
TC	GBLUP	0.51	0.52	0.53	0.54	0.54	0.54	0.59	0.59	0.54	0.58
	GBLUP + fixed PCs	–	–	–	–	–	0.57	0.61	0.61	0.55	0.60
	GBLUP + fixed-SNPs	–	–	–	–	–	0.58	0.64	0.64	0.61	0.64
	GBLUP + fixed-SNPs + fixed PCs	–	–	–	–	–	0.61	0.66	0.66	0.62	0.65

Marker Sets Include: Whole-Genome Sequence (WGS) data, LD Pruned SNP Sets (LD_2mil to LD_10k), and the optimum density of GWAS-Preselected Variants (TOP-BLINK, TOP-FarmCPU, TOP-MLMM, TOP-GLMM) for each GWAS-method-by-trait combination. Prediction models include: (1) Base GBLUP (without population structure control or fixed-effect SNPs), (2) GBLUP with a fixed-effect SNP (GBLUP + fixed SNP), (3) GBLUP with top six Principal Components as fixed effects (GBLUP + fixed PCs), and (4) GBLUP with both fixed-effect SNP and fixed PCs (GBLUP + fixed PCs + fixed SNP).

Results from the evaluation of marker density effects under the parental validation strategy using baseline GBLUP models revealed that predictive ability varied with density. Predictive ability ranged from 0.60 to 0.67 for FBC, 0.59 to 0.67 for AFW, 0.35 to 0.41 for FF, and 0.51 to 0.54 for TC (**Supplementary Table 5**). Across all traits, predictive ability generally increased with marker density but plateaued beyond LD_20k (~20,000 SNPs), indicating little gains at higher SNP densities. Models incorporating a GRM estimated from the lowest-density marker set (LD_10k) exhibited substantially lower predictive ability compared to those using higher-density marker sets (LD_20k to WGS), which showed only marginal variation in predictive ability among themselves. For TC, differences in predictive ability were relatively stable across marker densities, with a maximum difference of just 0.03 between LD_10k and WGS. Under the 5-fold CV, differences in predictive ability across marker densities were minimal for all traits (**Supplementary Table 7**).

#### 3.4.2 Effect of population structure on predictive ability

Incorporating the top six principal components (PCs) as fixed effects to account for population structure resulted in substantial reductions in predictive ability for all traits (**Table 2**). The decrease in predictive ability ranged from 0 to over 100% depending on marker set and validation approach employed, highlighting the dominant influence of population structure on genomic prediction within this gene-pool for these traits. Under parental validation, predictive ability based on WGS data decreased from 0.67 to 0.44 for FBC, from 0.67 to 0.30 for AFW, and from 0.41 to 0.30 for FF when population structure was accounted for (**Supplementary Table 6**). The exception was TC, where a slight increase in predictive ability was observed, rising from 0.54 to 0.57. Notably, while population structure correction reduced predictive ability for FBC, this decline was substantially mitigated when the FBC-associated SNP on chromosome 15 was fitted as a fixed effect in GBLUP models. When only the top six PCs were included as fixed effects, predictive ability for FBC dropped by 34%. However, when both the first six PCs and the most significant GWAS-identified SNP were jointly fitted as fixed effects, the reduction in predictive ability was mitigated to just 7%.

Similarly, results from 5-fold cross validation revealed a marked decline in predictive ability after correcting for population structure (**Supplementary Table 8**). However, unlike in the parental validation strategy where the predictive ability for TC remained stable despite population structure correction, the predictive ability in the 5-fold cross-validation declined sharply, dropping from 0.57 to 0.45 when using WGS data.

### 3.5 Genome-wide association studies

Utilizing three multi-locus GWAS approaches and one single-locus GWAS method on ~2 million SNPs, we identified 24 unique associations across three traits (**Table 3**): fruit blush color (FBC, n = 5; **Supplementary Figure 2**), average fruit weight (AFW, n = 11; **Supplementary Figure 3**), and trunk circumference (TC, n = 8; **Supplementary Figure 4**). Notably, the FBC-associated SNPs on chromosome 15 identified by the GLMM were in very strong LD with each other (mean *r^2^* = 0.94), forming a distinct peak. FarmCPU identified the most trait-associated SNPs among the four GWAS methods evaluated, identifying 20 significant associations, followed by BLINK (7), and the MLMM (2). In contrast, the GLMM only detected one association. For TC, all significant marker-trait associations were detected in trees assessed at 9 years of age, whereas no significant SNPs were identified in trees assessed at 12 years of age. The comparison of SNP positions with the annotated ‘Alphonso’ genome suggested that some SNPs were associated with regions containing putative loci for FBC, AFW, and TC previously identified in mango and other tree species (**Table 4**).

**Table 3:** Significant marker-trait associations for average fruit weight (AFW), fruit blush color (FBC), and trunk circumference (TC).

Trait	Marker name	Chr	Pos (bp)	P-value	MAF	GWAS method
AFW	NC_058139.1_14599216	3	14599216	2.19e-10	0.08	BLINK
	NC_058143.1_71757	7	71757	7.70e-10	0.3	BLINK
	NC_058151.1_3295704	15	3295704	1.42e-10	0.17	BLINK
	NC_058153.1_7169193	17	7169193	2.12e-20	0.13	BLINK, FarmCPU
	NC_058156.1_9929325	20	9929325	1.33e-10	0.37	BLINK
	NC_058138.1_17016987	2	17016987	1.78e-10	0.49	FarmCPU
	NC_058146.1_6357250	10	6357250	8.97e-09	0.09	FarmCPU
	NC_058149.1_10041368	13	10041368	1.99e-13	0.38	FarmCPU
	NC_058151.1_14147173	15	14147173	1.91e-11	0.11	FarmCPU
	NC_058153.1_2108313	17	2108313	2.10e-09	0.44	FarmCPU
	NC_058156.1_9195450	20	9195450	1.21e-10	0.27	FarmCPU
FBC	NC_058151.1_10729807	15	10729807	3.32e-22	0.35	BLINK, FarmCPU, GLMM
	NC_058140.1_17796415	4	17796415	1.92e-20	0.06	FarmCPU
	NC_058143.1_10454361	7	10454361	2.28e-09	0.46	FarmCPU
	NC_058143.1_15901033	7	15901033	1.87e-12	0.47	FarmCPU
	NC_058148.1_6029563	12	6029563	8.04e-10	0.18	FarmCPU
	NC_058151.1_10744410	15	10744410	4.12e-11	0.36	MLMM
TC	NC_058143.1_14357156	7	14357156	2.23e-14	0.35	BLINK, FarmCPU, MLMM
	NC_058137.1_13654943	1	13654943	5.37e-09	0.09	FarmCPU
	NC_058138.1_3215561	2	3215561	1.50e-09	0.36	FarmCPU
	NC_058138.1_9666205	2	9666205	2.04e-08	0.07	FarmCPU
	NC_058139.1_20457585	3	20457585	7.01e-11	0.16	FarmCPU
	NC_058148.1_14363648	12	14363648	1.66e-10	0.19	FarmCPU
	NC_058149.1_5010618	13	5010618	7.26e-10	0.16	FarmCPU
	NC_058154.1_7179850	18	7179850	2.44e-10	0.12	FarmCPU

Table legend: The table displays trait, marker name, chromosome (Chr), position (Pos) in base pairs, GWAS-derived p-value, minor allele frequency (MAF) of the trait-associated SNP, and GWAS Method.

**Table 4:** Candidate genes identified near significant SNP markers associated with fruit blush color (FBC), average fruit weight (AFW), and trunk circumference (TC) in mango.

Trait	Chr	MAF	Distance from SNP (kb)	Candidate gene	Functional role	Reference
FBC	15	0.35	0.52 kb	MYB114-like transcription factor	Fruit coloration	(Kanzaki et al., 2020; Plunkett et al., 2019)
AFW	2	0.49	158 kb	Cell division control protein	Fruit size	(Devoghalaere et al., 2012; Karim et al., 2022; Zhang et al., 2006)
AFW	7	0.30	110 kb	Two cell division control proteins	Fruit size	(Devoghalaere et al., 2012; Karim et al., 2022; Zhang et al., 2006)
AFW	13	0.38	12 kb	Two auxin response factors	Fruit size	(Devoghalaere et al., 2012)
AFW	13	0.38	26 kb	Ethylene-responsive transcription factor	Fruit size	(Bally et al., 2021)
AFW	15	0.17	33 kb	GDSL esterase/lipase	Fruit size	(Bally et al., 2021)
AFW	15	0.17	160 kb	Cell number regulator	Fruit size	(Devoghalaere et al., 2012)
TC	2	0.07	21 kb	Growth regulating factor gene	Tree trunk diameter	(Wu et al., 2021)
TC	2	0.36	6 kb and 16 kb	Two auxin efflux carrier genes	Tree growth	(Qi et al., 2020; Zhang et al., 2015)
TC	7	0.35	68 kb	GATA transcription factor	Tree growth	(An et al., 2014)

Candidate genes were identified based on alignment with the annotated ‘Alphonso’ reference genome. The Table lists the associated Trait, Chromosome (Chr), minor allele frequency (MAF) of the trait-associated SNP, distance between the SNP and candidate gene, the candidate gene or transcription factor, its functional role, and supporting references where the gene or transcription factor’s role has previously been reported.

#### 3.5.1 Genotype and GEBVs relationship

Reliable trait-associated SNPs (identified by at least two GWAS methods) showed clear effects on phenotypic variation, as revealed by GEBVs for the three genotypic classes: homozygous reference, heterozygous, and homozygous alternate allele (**Supplementary Figure 5**-**Supplementary Figure 7**). For FBC, the SNP on chromosome 15 (G/A) showed that cultivars with the GG genotype (e.g., ‘Ah Ha!’, ‘Tommy Atkins’, and ‘Irwin’) had significantly higher FBC ratings (*p* < 0.0005, mean GEBV = 2.0) compared to those carrying the A allele either in homozygous form (mean GEBV = 1.2; e.g., ‘Dashehari’, ‘Mallika’, and ‘Arumanis A’) or heterozygous form (mean GEBV = 1.0; e.g., ‘Maha Chanook’, ‘Alphonso’, and ‘Carabao Pep’). For AFW, the SNP on chromosome 17 (A/G) revealed that cultivars with the A allele in homozygous form had significantly lower fruit weight (p< 0.0005; mean GEBV = 322.0 g) than the heterozygous cultivars (mean GEBV = 415.2 g). For TC, the SNP on chromosome 7 (T/A) revealed that AA genotypes (e.g., ‘Manjeera’, ‘Lippens’) had significantly lower trunk circumference (*p* < 0.0005; mean GEBV = 45.5 cm) compared to cultivars carrying the T allele either in homozygous form (mean GEBV = 56.5 cm) or heterozygous form (mean GEBV = 52.4 cm). Notably, heterozygous (T/A) genotypes also had significantly smaller trunk circumference (*p* < 0.0005) than homozygous TT genotypes.

### 3.6 Incorporation of GWAS results in GBLUP models

#### 3.6.1 Preselected variants from GWAS increased predictive ability

**Figure 2:**
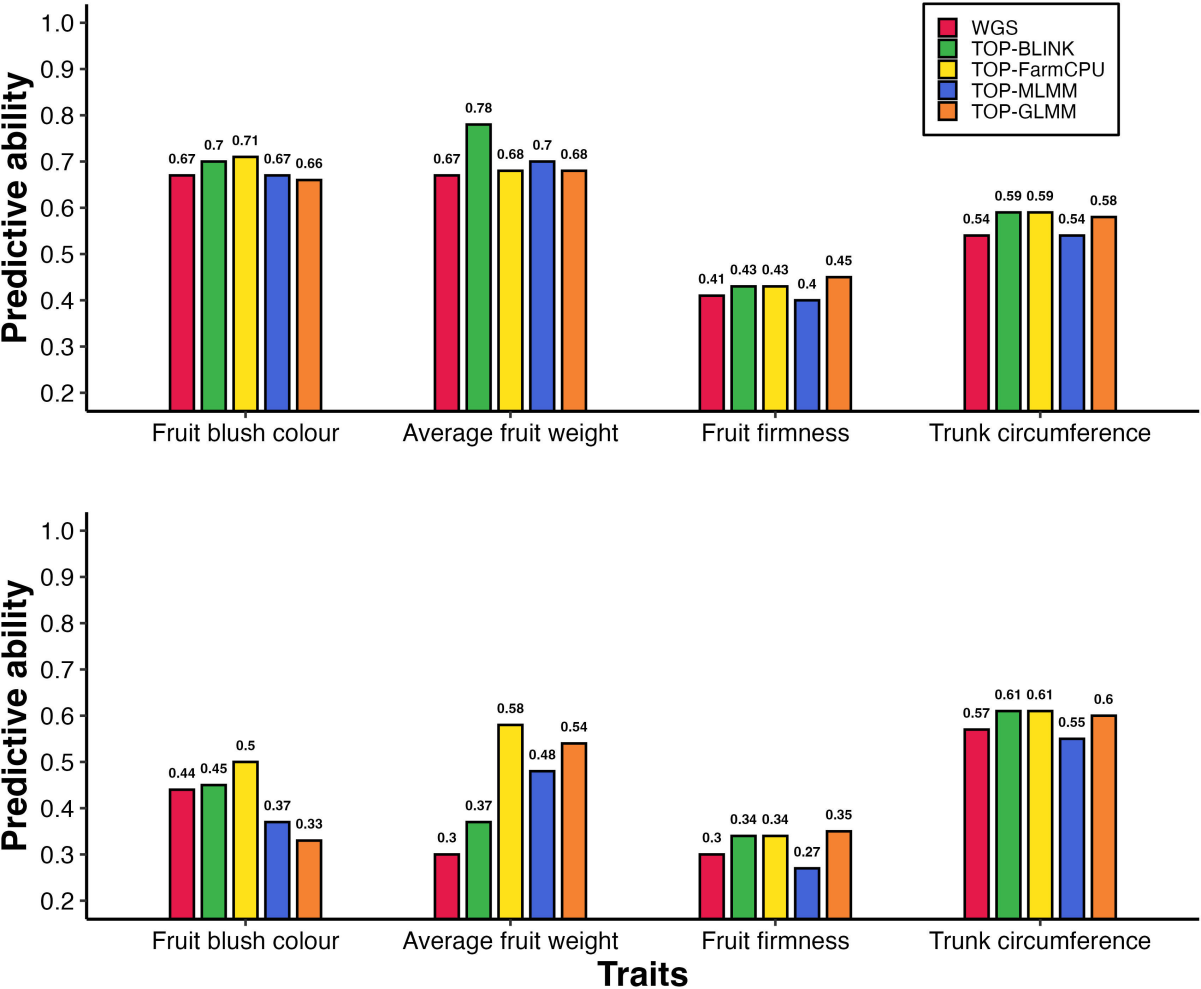
Predictive ability of breeding population parent phenotypes: **(A)** without accounting for population structure and **(B)** while accounting for population structure, using models without fixed-effect SNPs. Bars represent predictive abilities across marker sets: WGS data and GWAS-preselected variants (TOP-BLINK, TOP-FarmCPU, TOP-MLMM, TOP-GLMM). Notably, under scenario A, predictive ability for AFW increased from 0.67 to 0.78 when 20,000 TOP-BLINK SNPs were used instead of WGS data.

**Figure 3:**
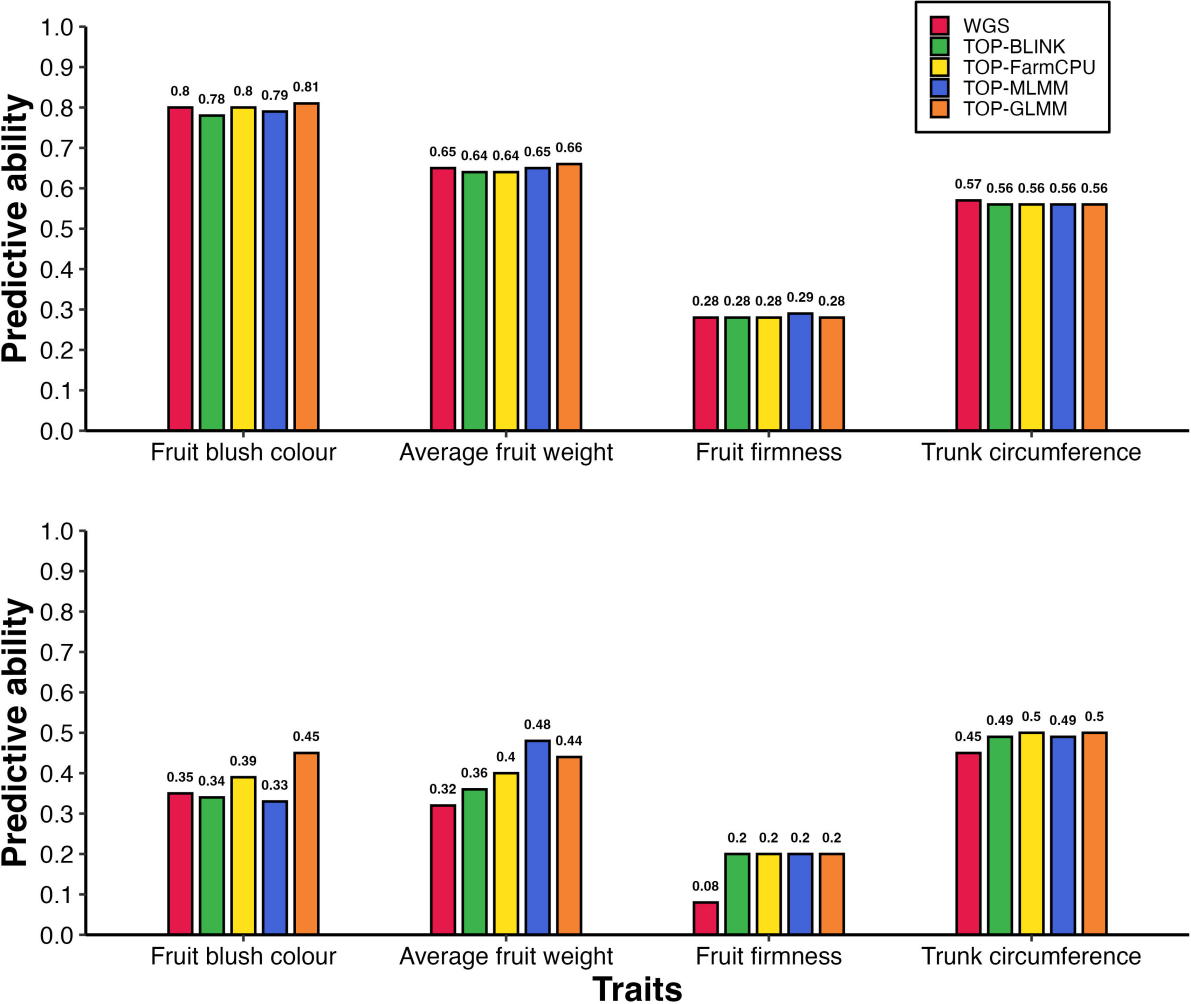
Predictive ability of gene pool individuals under 5-fold cross-validation: **(A)** without accounting for population structure and **(B)** with population structure correction, using models without fixed-effect SNPs. Bars represent predictive abilities across different marker sets, including WGS data (WGS) and GWAS-preselected variants (TOP-BLINK, TOP-FarmCPU, TOP-MLMM, TOP-GLMM). Notably, under scenario B, predictive ability for AFW increased from 0.32 to 0.48 when 1,000 TOP-FarmCPU SNPs were used instead of WGS data.

Models incorporating a GRM derived from variants preselected based on the highest ranked probability of effect as estimated using GWAS improved predictive ability across all traits, with improvements of up to 93% under parental validation (**Table 2**). The magnitude of these improvements varied depending on the trait, density of GWAS-preselected variants, GWAS method applied, and whether population structure was accounted for. When using base models (i.e., models without population structure correction or fixed-effect SNPs) under the parental validation strategy, preselecting variants based on GWAS showed an advantage depending on the GWAS method used to identify variants, particularly for AFW and to a lesser extent for FBC, FF, and TC (**Figure 2A**). The predictive ability for AFW was markedly higher when using 20,000 TOP-BLINK GWAS-preselected variants, reaching 0.78, compared to 0.67 using the complete WGS dataset. In contrast, improvements in predictive ability for other traits were more modest, increasing from 0.67 to 0.71 for FBC using 100,000 SNPs from the TOP-FarmCPU set, from 0.54 to 0.59 for TC using either 20,000 or 50,000 SNPs from the TOP-BLINK or TOP-FarmCPU set, and from 0.41 to 0.45 for FF using 1,000 SNPs from the TOP-GLMM set. However, under 5-fold cross-validation using models that did not account for population structure, GWAS-based SNP preselection did not lead to improvements in predictive ability across any of the traits (**Figure 3A**, **Supplementary Table 7**).

The increases in predictive ability observed with GWAS-preselected variants relative to WGS data were much larger when population structure was accounted for (**Figure 2B**). Under the parental validation strategy, adjusting for population structure in GBLUP models led to a 93% improvement in predictive ability for AFW, increasing from 0.30 to up to 0.58 when 15,000 variants from the TOP-FarmCPU set were used instead of WGS data. Similar improvements in predictive ability were observed for FBC and FF, rising from 0.44 to 0.50 using either 50,000 or 100,000 TOP-FarmCPU SNPs for FBC, and from 0.30 to 0.35 using top 1,000 SNPs selected by GLMM for FF. In contrast, there was little variation in predictive ability for TC between models that included GWAS pre-selected variants with or without adjustment for population structure.

A comparable pattern was observed under the 5-fold cross-validation strategy in GBLUP models that included population structure correction (**Figure 3B**). Specifically, predictive ability increased by up to 29% for FBC (from 0.35 to 0.45), 50% for AFW (from 0.32 to 0.48), and 150% for FF (from 0.08 to 0.20), while TC showed a modest improvement of 11% (from 0.45 to 0.50). The highest predictive abilities under 5-fold cross validations were achieved using 10,000 SNPs from TOP-GLM for FBC, 1,000 SNPs from TOP-MLMM for AFW, 1,000 SNPs from all GWAS methods for FF, and 10,000 or more SNPs from either TOP-FarmCPU or TOP-GLMM for TC (**Supplementary Table 8**). Notably, for FF and TC, the predictive abilities obtained using GWAS-preselected variants were comparable to those achieved using LD-pruned marker sets (LD_10k to LD_2mil).

Predictive abilities using GWAS-preselected variants showed substantial variation depending on marker density and validation strategy, with no consistent trend across traits (**Supplementary Tables 5**, **7**). Under the parental validation strategy in models ignoring population structure, the highest predictive abilities were achieved using 20,000 SNPs from BLINK for AFW, 100,000 SNPs from FarmCPU for FBC, 1,000 SNPs from GLMM for FF, and either 20,000 or 50,000 SNPs from BLINK or FarmCPU for TC. In contrast, under 5-fold cross validation, predictive abilities remained relatively stable across different marker densities (**Supplementary Table 7**). Differences in maximum predictive ability between GWAS models were generally small (< 0.03), except for FBC and AFW in models that accounted for population structure (**Supplementary Table 8**). In these cases, the highest predictive abilities were achieved using 10,000 and 1,000 SNPs from TOP-GLM and TOP-MLMM, respectively. All subsequent results are based on the parental validation strategy using both the full WGS dataset and the optimal set of GWAS-preselected variants for each trait.

#### 3.6.2 Fixed-effect SNPs increased predictive ability for fruit blush color and trunk circumference

**Figure 4:**
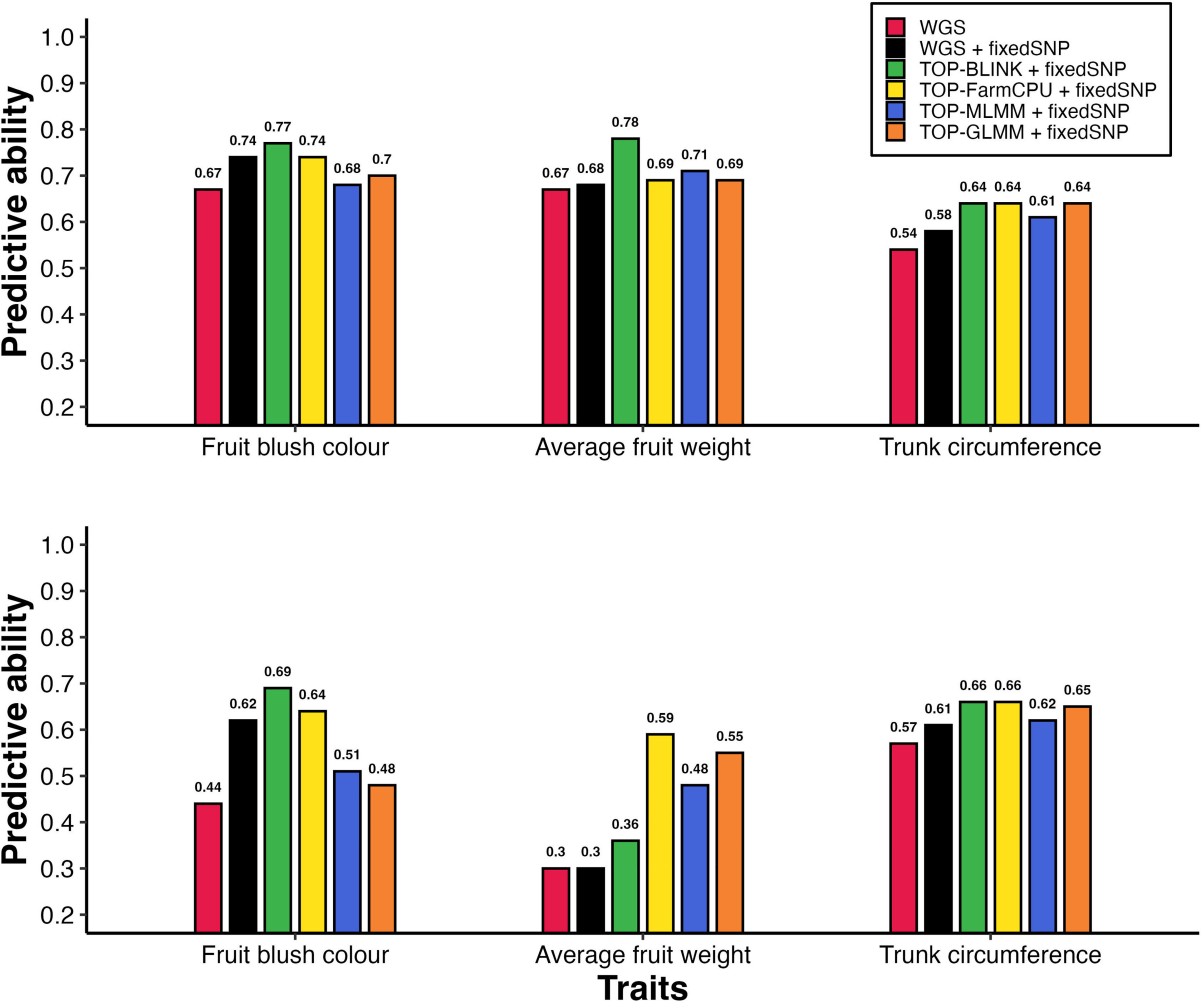
Predictive ability of breeding population parent phenotypes: **(A)** without population structure control and including fixed-effect SNPs, and **(B)** with population structure correction and fixed-effect SNPs. Bars represent predictive abilities across marker sets: WGS data (WGS), WGS with fixed-effect SNP (WGS + fixedSNP), and GWAS-preselected variants with fixed-effect SNP (TOP-BLINK + fixedSNP, TOP-FarmCPU + fixedSNP, TOP-MLMM + fixedSNP, TOP-GLMM + fixedSNP). Notably, under scenario B, predictive ability for FBC increased from 0.44 to 0.69 when using 50,000 TOP-BLINK SNPs with fixed-effect SNP instead of using WGS data alone.

The impact of incorporating reliable markers as fixed effects on predictive ability varied depending on the trait, marker set, and whether population structure was accounted for (**Figure 4**, **Supplementary Table 9**, **Supplementary Table 10**). Our findings indicate that incorporating a reliable SNP as a fixed effect in prediction models markedly improved predictive ability for FBC and TC, with gains of up to 0.26 and 0.07, respectively.

For FBC, incorporating the reliable trait-associated SNP on chromosome 15 as a fixed effect in GBLUP models without accounting for population structure resulted in an improvement in predictive ability ranging from 0.01 to 0.07 compared to models without the fixed-effect SNP. Notably, predictive ability increased from 0.67 to 0.74 with WGS data and from 0.70 to 0.77 using 100,000 TOP-BLINK markers when the FBC-reliable marker was included as a fixed effect. Strikingly, under population structure correction, the enhancement in predictive ability due to the inclusion of the FBC-reliable marker as a fixed effect was even more pronounced, with gains ranging from 0.12 to 0.26. In these population structure corrected models, predictive ability increased from 0.44 to 0.62 with WGS data, from 0.45 to 0.69 using 50,000 TOP-BLINK markers, from 0.50 to 0.62 using either 50,000 or 100,000 TOP-FarmCPU markers, from 0.37 to 0.51 using 15,000 TOP-MLMM markers, and from 0.33 to 0.48 using 10,000 TOP-GLMM markers. Further analysis using ~ 2 million SNPs showed that this FBC-associated SNP accounted for 36% of the genetic variance (results not shown).

Incorporating the reliable TC-associated SNP on chromosome 7 as a fixed effect in GBLUP models also improved predictive ability, with gains of up to 0.07 in models without population structure control, and up to 0.06 when population structure was accounted for. For example, predictive ability increased from 0.54 to 0.58 with WGS data, from 0.59 to 0.64 using 20,000 or 50,000 SNPs from either BLINK or FarmCPU, and from 0.54 to 0.61 with 20,000 TOP-MLMM markers when the reliable TC-associated SNP was included as a fixed effect in models without population structure correction. A similar pattern was observed when population structure was accounted for, with the predictive ability for WGS data increasing from 0.57 to 0.61, from 0.61 to 0.66 using 50,000 SNPs from either BLINK or FarmCPU, and from 0.55 to 0.62 using 15,000 TOP-MLMM markers. In contrast, for AFW, adding fixed-effect markers to the prediction models did not improve predictive ability.

#### 3.6.3 Improved prediction via combined use of GWAS-preselected variants and fixed-effect SNPs

Combining GWAS-preselected variants with fixed-effect SNPs substantially improved predictive ability for FBC and TC compared to models using WGS data alone or GWAS-preselected variants alone. The highest predictive abilities for these traits were achieved using this integrated approach both with and without population structure correction (**Table 2**; **Figure 4**). For example, substituting WGS data with TOP-BLINK markers improved predictive ability for FBC from 0.67 to 0.70 (**Figure 2A**). Incorporating the FBC-associated reliable SNP on chromosome 15 as a fixed effect increased predictive ability with WGS data from 0.67 to 0.74 (a 0.07 increase). Notably, combining 100,000 GWAS-preselected variants from BLINK with the fixed-effect SNP yielded a substantial improvement, boosting predictive ability by 0.10 (from 0.67 with WGS data to 0.77 using a combination of GWAS-preselected variants and the fixed-effect SNP). A similar trend was observed when population structure was accounted for, with the highest predictive ability (0.69) achieved by incorporating the FBC-reliable SNP as a fixed effect in a GBLUP model based on a GRM derived from 50,000 TOP-BLINK markers. This predictive ability represents a substantial improvement, exceeding that of WGS data alone by 0.25 and that of TOP-BLINK markers alone by 0.24, and surpassing WGS data with a fixed-effect SNP by 0.07.

A similar trend was observed for TC, where the highest predictive abilities were achieved by integrating GWAS-preselected variants with fixed-effect SNPs in a single GBLUP model. Specifically, including 20,000 or 50,000 GWAS-preselected variants from TOP-FarmCPU or TOP-BLINK alongside fixed-effect SNPs improved predictive ability by 0.1, increasing from 0.54 with WGS data to 0.64. This enhancement in predictive ability surpasses the gains of 0.06 and 0.05 obtained when using either WGS data plus fixed-effect SNPs or GWAS-preselected variants alone. Notably, a comparable pattern emerged in GBLUP models that accounted for population structure, with predictive abilities remaining nearly identical to those observed in models without population structure correction.

When considering the optimal marker density for GWAS-preselected variants under parental validation, defined as the density yielding the highest predictive ability, TOP-BLINK and TOP-FarmCPU both achieved the highest predictive abilities in eight of the fourteen trait-by-scenario combinations (four traits and four scenarios [GBLUP, GBLUP + fixed SNP, GBLUP + fixed PCs, and GBLUP + fixed PCs + fixed SNP]). TOP-GLMM produced the highest predictive ability in three combinations, while MLMM did not result in the highest predictive ability in any of the scenarios. In contrast, the performance of GWAS models under 5-fold cross-validations was comparable across all traits when population structure was ignored. However, under models that accounted for population structure, GWAS-preselected variants identified using the MLMM and GLMM yielded the highest predictive ability for AFW and FBC, respectively.

## 4 Discussion

### 4.1 Effective population size and linkage disequilibrium

Our results indicate that estimates of effective population size (*Ne*) in the QMBP gene-pool collection (*Ne* = 129, excluding accessions from Southeast Asia) and the parental population (*Ne* = 104) are well above the recommended minimum of 50 required to minimize short-term inbreeding (Clarke et al., 2024). These large estimates of *Ne* indicate a high number of independently segregating chromosome segments, suggesting that high density marker sets are needed to ensure marker-QTL LD for accurate genomic prediction (Grattapaglia, 2014). The estimates of *Ne* in both the gene-pool collection and parental population suggest that these populations maintain sufficient genetic diversity to sustain long-term genetic gains (White et al., 2007) in the QMBP.

Mango is an outcrossing and highly heterozygous species (Wilkinson et al., 2022) and thus would be expected to have rapid LD decay (Vos et al., 2017). The rapid LD decay observed in our study likely reflects the substantial genetic diversity within the gene-pool collection (Wilkinson et al., 2022), in agreement with the high *Ne* estimates. Specifically, LD decay of *r^2^* = 0.2 (the commonly considered minimum LD threshold for accurate genomic prediction) occurred at 3.6 kb in our study using WGS data. This is comparable to estimates in other outcrossing species like *Eucalyptus* (4 kb; Butler et al., 2022) and *Populus* (3–6 kb; Slavov et al., 2012) but lower than reported in a diverse historical apple population (0.1 kb; Migicovsky et al., 2016). The rapid LD decay observed in this study should increase the resolution of GWAS studies by allowing for accurate identification of causal variants. This improvement stems from the presence of short haplotype blocks which mitigate the confounding effects of strong LD between causal mutations and numerous non-causal loci, thereby reducing the noise-to-signal ratio and improving GWAS resolution (Jang et al., 2023b). The mean *r^2^* between adjacent WGS SNPs in our study (0.33) is comparable to values reported in apple (0.32; Kumar et al., 2012) and pear (0.33; Minamikawa et al., 2018), indicating a strong potential for implementing genomic selection in mango.

### 4.2 Genome-wide association studies

#### 4.2.1 Fruit blush color

This study identified five distinct and statistically significant associations for FBC (**Table 3**, **Supplementary Figure 2**). Notably, a MYB114 transcription factor was located just 0.5 kb from a key FBC-associated marker on chromosome 15, consistently identified by three different GWAS methods. MYB transcription factors are widely reported to regulate fruit skin color in multiple fruit tree species, including mango (Kanzaki et al., 2020; Wilkinson et al., 2025), apple (Plunkett et al., 2019; Sun et al., 2021), pear (Cong et al., 2021; Zhang et al., 2021) and kiwifruit (Ampomah-Dwamena et al., 2019). These genes are central regulators of the anthocyanin biosynthesis pathway, which plays a critical role in pigmentation of fruit peels (Gao et al., 2021). The well-established role of anthocyanin accumulation in contributing to red skin coloration in fruits is consistent with previous findings in mango. Wang et al. (2020) demonstrated that anthocyanin biosynthesis genes were significantly upregulated in the peel of red-skinned mango cultivars compared to yellow- or green-skinned types. Additionally, Kanzaki et al. (2020) reported that exposure to light stimulus increased the expression of *MiMYB1* and *MiMYB4* transcription factors in reddened mango fruit, further highlighting the involvement of MYB transcription factors and light exposure in regulating peel coloration.

#### 4.2.2 Fruit weight

This study identified 11 novel SNPs significantly associated with AFW (**Table 3**, **Supplementary Figure 3**), a key trait for influencing consumer appeal and market value, and therefore a major target for improvement in mango breeding programs (Bally et al., 2021). Our results support the role of hormone-mediated cell division in determining fruit weight, consistent with findings in other horticultural fruit tree species (Karim et al., 2022; Li et al., 2024; Zhang et al., 2006). Notably, two auxin response factors were identified within 12 kb of an AFW-associated SNP on chromosome 13, suggesting a likely regulatory role of auxin signaling in fruit weight variation. Auxin response factors have previously been implicated in apple fruit weight variation through modulation of cell division and expansion (Devoghalaere et al., 2012). Additionally, an AFW-associated SNP on chromosome 7 was located ~110 kb from two cell division control proteins, reinforcing the mechanistic link between cell division during mango fruit development and fruit size. Similar associations have been reported in sweet cherry (*Prunus avium* L.), where a fruit size QTL was closely linked to a gene governing cell number, underscoring the conserved nature of these genetic mechanisms across species (De Franceschi et al., 2013).

#### 4.2.3 Trunk circumference

We identified eight unique marker-trait associations for TC across seven chromosomes (**Table 3**, **Supplementary Figure 4**). Our analyses identified a GATA transcription factor located within 70 kb of a TC-associated SNP on chromosome 7. GATA transcription factors have been reported to regulate tree growth in *Populus* (An et al., 2020). BLAST analysis revealed that the GATA transcription factor identified in our study shares 86% sequence similarity with the one reported in *Populus* (An et al., 2020), suggesting similar regulatory mechanisms in mango tree growth. GATA transcription factors are known to modulate the expression of auxin efflux carrier genes, facilitating the basipetal movement of auxins to the roots (An et al., 2020, An et al., 2014). In our study, two auxin efflux carrier genes were located just 6 kb and 16 kb from TC-associated SNPs on chromosome 2, further supporting the potential regulatory role of auxin transport in mango tree growth.

Prior studies strongly support a model in which plant dwarfism results from reduced expression of PIN genes (auxin efflux carriers) in stem bark tissues, leading to impaired auxin transport to the roots. This disruption limits root growth and cytokinin biosynthesis, ultimately constraining shoot development (An et al., 2017; Li et al., 2018). These mechanisms align with previous studies in apple, where use of dwarfing inter-stock (M9) led to decreased expression of auxin efflux carrier genes in stem bark tissues, suppressing the basipetal movement of auxins and leading to reduced root and shoot development (Zhang et al., 2015). Similar findings in pear demonstrated significantly higher expression levels of the *PcPIN-L* auxin efflux carrier gene in standard-size trees compared to dwarf types (Qi et al., 2020), further reinforcing the role of auxin transport in tree growth regulation.

In addition to the GATA transcription factor and auxin efflux carrier proteins, we identified a growth-regulating factor gene located approximately 21 kb from a TC-associated SNP on chromosome 2. This, together with the proximity of auxin efflux carrier genes and the GATA transcription factor to TC-associated SNPs, suggests the involvement of a coordinated regulatory network governing tree growth in mango. The trait-associated markers identified for FBC, AFW and TC in this study represent a valuable resource for marker-assisted breeding in mango, pending validation in independent populations.

#### 4.2.4 Multi-locus GWAS are powerful at detecting trait-associated SNPs

Our findings underscore the superior statistical power of multi-locus GWAS methods compared to single-locus approaches. Consistent with previous studies (Cebeci et al., 2023; Huang et al., 2019; Minamikawa et al., 2018), multi-locus GWAS methods, particularly BLINK and FarmCPU, identified more significant marker-trait associations than the single-locus GWAS approach (GLMM). The increased power of multi-locus GWAS stems from their ability to account for LD between SNPs (as in BLINK) while simultaneously testing multiple markers, enhancing the detection of small-effect loci associated with a trait (Segura et al., 2012; Wang et al., 2016). BLINK and FarmCPU, which use multi-locus strategies and iterative inclusion of pseudo-QTNs, tend to capture both large- and small-effect loci more robustly, especially in polygenic traits. In contrast, MLMM’s stepwise regression approach appears to underperform, likely due to over-adjustment for population structure and the inherent sensitivity of its sequential covariate inclusion, which may mask genuine signals.

Our results, particularly for AFW where BLINK and FarmCPU identified more significant marker-trait associations, highlight the value of this multi-method GWAS strategy. These findings are consistent with reports by Minamikawa et al. (2018) and Kumar et al. (2019), who identified a higher number of trait-associated SNPs in pears (*Pyrus pyrifolia*) by employing multiple GWAS methods rather than relying on a single approach. Integrating results across multiple GWAS methods is a powerful strategy to identify additional marker-trait associations as no single method is optimal for all traits. Moreover, loci detected by the different methods do not completely overlap (Zhou et al., 2023). The use of a combination of complementary GWAS methods not only strengthens statistical robustness but also strengthens confidence in associations consistently detected across analyses, making these associations strong candidates for marker development and functional validation.

### 4.3 Genomic prediction

#### 4.3.1 Simply increasing marker density to WGS level does not increase predictive ability

In our study, we observed that increasing marker density beyond a certain threshold, even up to WGS level, did not yield further improvements in predictive ability (**Table 2**). These findings are consistent with previous studies (Bedhane et al., 2021; Moghaddar et al., 2019; Raymond et al., 2018b, Raymond et al., 2018c; van Binsbergen et al., 2015; VanRaden et al., 2017) that also found little or no improvement in prediction accuracy when using WGS variants compared to lower density or high-density SNP chips. A plausible explanation is that WGS data include many variants that are not in strong LD with the causative loci (van Binsbergen et al., 2015). These non-informative markers may not capture the QTL effects or accurately reflect genetic relationships at causal loci, potentially undermining the performance of GP models through over-shrinkage of QTL effects. This phenomenon likely reflects the balance between capturing true causal variation and overfitting to random, non-informative variation. Our analyses using different LD-pruned subsets (e.g. LD_2mil, LD_800k, etc.) indicated that predictive ability tended to plateau or even decline when the number of markers exceeded an optimal threshold. This threshold is inherently linked to the underlying LD structure and genetic architecture of the trait in question.

#### 4.3.2 Marker preselection could enhance genomic predictive ability

This study showed that GRMs constructed using GWAS-preselected variants resulted in higher predictive abilities across the four studied traits compared to GRMs built using all WGS variants (**Table 2**, **Figures 2**, **3**). These findings highlight that preselecting WGS markers likely to be in LD with causal mutations, while excluding those that do not capture genetic relationships at causal loci, can improve genomic predictive ability. Thus, it appears that including markers not in LD with causative mutations in GRM construction may cause the realized genetic relationships to diverge from true relationships at causal loci, thereby reducing the performance of GBLUP models. However, when markers preselected for their potential causal effects are used, the GRM is dominated by SNPs in high LD with QTL for the target trait. Thus, the trait-specific GRM may better capture the genetic relationships among individuals at unobserved causal loci, potentially enhancing the accuracy of genomic predictions. Our results are consistent with those of Tan and Ingvarsson (2022) who showed that when the top 1% of markers from GWAS are selected, the accuracy of genomic predictions can be increased significantly. Chen et al. (2023) also showed that performing GP using a GRM built using 100 preselected markers resulted in improved prediction accuracies compared to models based on all markers.

While our results clearly demonstrate that the integration of GWAS-preselected variants improves predictive ability, we acknowledge that validation confined to a single, relatively small dataset may limit the external applicability and generalizability of our findings. Such internal validation alone does not adequately account for potential biases introduced by population-specific genetic structure or unique environmental factors. Although we employed a 5-fold cross-validation strategy to strengthen robustness of our model assessment, external validation in large, independent datasets such as a full-sib population remains essential. Such validation would verify whether the observed improvement in predictive performance genuinely reflects enhanced capture of causal genetic variation.

#### 4.3.3 Fixed-effect SNPs improve predictive ability

While the use of GWAS-preselected variants increased genomic predictive ability in our analyses, this approach still suffers from the assumption of the GBLUP model that all markers contribute an equal and individually small proportion of the total genetic variance (Meuwissen and Goddard, 2010). However, increasing evidence supports the hypothesis that SNPs in high LD with causal mutations explain more genetic variance than those in low LD (Meuwissen et al., 2024). Incorporating fixed-effect SNPs into GBLUP models appeared to improve predictive ability for both FBC and TC, likely by capturing variation associated with major QTLs (**Figure 4**). This strategy enabled us to account explicitly for the effects of markers with large estimated effects, potentially helping to separate their contribution from those assumed under the infinitesimal model. While these results suggest benefits from including such markers, it remains important to recognize that the identified SNPs may not represent true causal variants, and further validation in an independent population such as a full-sib family would be needed to confirm their functional significance. The differentiation between large- and small-effect QTLs appears to model better the true genetic architecture of traits, leading to more accurate prediction models. This is especially true when markers in LD with major genes are treated as fixed effects (Li et al., 2019). Our findings are consistent with prior studies. For example, Kostick et al. (2023) demonstrated a substantial improvement in the predictive ability of ‘percent red overcolor’ in apple, which increased from 0.33 to 0.80 upon inclusion of a fixed-effect SNP at a fruit color locus. Similarly, Nsibi et al. (2020) reported a 25.8% increase in prediction accuracy for apricot (*Prunus armeniaca*) fruit color (hue angle) after incorporating two major QTLs as fixed effects.

Critically, the effectiveness of using fixed-effect SNPs relies on their LD with a QTL, as reported by Li et al. (2019). In this study, the fixed-effect SNPs that enhanced predictive abilities were consistently identified by three GWAS methods (reliable SNPs), strengthening the evidence that these SNPs are likely in LD with underlying QTLs.

#### 4.3.4 Combining preselected variants and fixed-effect SNPs further enhances predictive ability

In our study, we demonstrated that while the utilization of GWAS-preselected variants or fixed-effect SNPs can enhance predictive ability, further improvements can be achieved through the integration of preselected variants with fixed-effect SNPs (**Table 2**, **Figure 4**). Traditional GBLUP models employing a single GRM constructed from GWAS-preselected variants do not fully capitalize on the predictive potential of large-effect SNPs due to the inherent assumptions of the infinitesimal model, which overly constrains their contribution to the total genetic variance. By contrast, our approach, combining preselected variants and fixed-effect SNPs, benefits from more accurate estimation of genomic relationships at causative loci. If all markers explain the same proportion of the total genetic variance, as is the assumption of the infinitesimal model, there would be no notable reduction in heritability when significant SNPs from GWAS are fitted as fixed effects in GBLUP models. However, our analyses demonstrated a notable reduction in additive genetic variance due to the anonymous markers and heritability when the fixed-effect SNP for FBC was included in GBLUP models, suggesting that a substantial portion of the additive genetic variance was explained by this SNP potentially due to its LD with the causative mutation. For mango breeding, fixed SNPs associated with FBC and TC provide particularly strong gains in predictive ability and should be prioritized for marker-assisted prediction pipelines.

While our findings demonstrate that integrating GWAS-preselected variants with fixed-effect SNPs can enhance genomic predictive ability, several limitations warrant discussion. First, the relatively modest training population size used in our study may limit statistical power to detect small-effect loci and increase the risk of overfitting, raising concerns about the external validity of this approach. Additionally, the specific population structure of our study may not fully represent the broader genetic landscape of mango germplasm, potentially affecting the transferability of our findings to more diverse populations. If between-subpopulation genetic variance differs across populations, the benefits of marker preselection and fixed-effect SNP integration may not be universally applicable. Future studies should validate these results in larger, independent datasets and assess the approach’s robustness across different genetic backgrounds to ensure broader applicability.

Several inconsistencies in predictive ability across varying densities of GWAS-preselected SNPs and different GWAS models highlight the practical challenges of selecting an appropriate GWAS method for variant preselection and determining the optimal number of SNPs to include. Such inconsistencies have important downstream implications, as the choice of GWAS method and preselected variants directly influences the construction of the GRM and the inclusion of fixed-effect SNPs in prediction models, ultimately affecting prediction accuracy. To address these inconsistencies and leverage the complementary strengths of individual GWAS methods, an ensemble-based approach that aggregates summary statistics from multiple GWAS models may offer a more robust solution. Such an approach could combine p-values, effect sizes, or marker rankings to prioritize SNPs that are consistently identified across methods, thereby balancing both sensitivity and specificity. Although ensemble GWAS has primarily been applied to the identification of causative variants (Zhou et al., 2023), its potential for SNP preselection in genomic prediction remains untapped. Meanwhile, ensemble genomic prediction models which aggregate predictions from multiple methods, have demonstrated improved accuracy in maize (Tomura et al., 2025), common bean (Chiaravallotti et al., 2025), and across cattle, wheat, and human datasets (Gu et al., 2024), underscoring the potential of model integration at various stages of the genomic prediction pipeline. While ensemble GWAS remains underexplored, a practical strategy for breeders is to prioritize markers consistently identified across multiple GWAS methods and benchmark the resulting models through cross-validation. This ensures that selected SNPs are both reproducible and practically useful in applied breeding programs.

#### 4.3.5 Multi-locus GWAS are powerful approaches for variant preselection

Our findings demonstrate that the predictive ability of models based on GWAS-preselected variants varies depending on the GWAS methodology employed. The superior performance of BLINK and FarmCPU compared to MLMM and the GLMM indicates their greater power in ranking markers based on LD with QTLs, thereby enabling the selection of more informative SNPs for genomic prediction. Beyond detecting a higher number of trait-associated SNPs than the MLMM and GLMM, these methods likely provide a more refined prioritization of markers with strong trait relevance. This superior performance can be attributed to their ability to effectively eliminate confounding effects between testing markers and both population structure (Q) and kinship (K) by dividing the multi-locus linear mixed model (MLMM) into components using either a fixed-effects model (FEM) and a random effects model (REM, pseudo-QTNs) in FarmCPU, or a fixed-effects model (FEM, for selecting pseudo-QTNs) and Bayesian Information Criterion (BIC) in BLINK (Huang et al., 2019; Liu et al., 2016). The use of pseudo-QTNs selected using REM in FarmCPU and FEM in BLINK as covariates effectively control false positives while retaining power to detect true associations. These features likely increase the probability of detecting SNPs that surpass the Bonferroni threshold as well as prioritizing biologically informative variants for use in genomic prediction.

The observation that, in some cases, differences in predictive ability across GWAS methods and varying densities of preselected SNPs were minimal suggests possible redundancy among SNP sets, shared association signals across GWAS methods, or the inherently polygenic architecture of the traits. One possible explanation is that methods such as BLINK and FarmCPU initially fit a general linear model (GLM), and when no significant associations are detected, they may default to reporting GLM results (Zhiwu Zhang, personal communication). This can result in overlapping sets of preselected SNPs across methods, which may explain the similar or comparable predictive abilities observed among BLINK, FarmCPU, and the GLMM for fruit firmness and trunk circumference under parental validation. A second contributing factor to the minor differences in predictive ability may be the presence of shared association signals across GWAS methods, where overlapping SNPs are selected due to consistently low p-values, suggesting potential relevance to the trait despite not reaching strict statistical significance. A third contributing factor is marker redundancy, which may occur even when the sets of GWAS preselected variants differ, if the SNPs are in LD and tag the same underlying QTLs. As a result, different sets of preselected SNPs may contribute similar genetic information to the prediction model, resulting in minimal variation in predictive ability. These modest differences are also consistent with the polygenic architecture of fruit quality traits and tree growth, where predictive ability is distributed across many loci rather than being driven by a few large-effect variants (Dong et al., 2024; Srivastav et al., 2023).

#### 4.3.6 Accounting for population structure reduces predictive ability in mango gene-pool

Our analysis revealed a marked decline in predictive ability when population structure was accounted for in prediction models (**Figures 2**, **3**), a pattern consistent with that reported by Guo et al. (2014) for wheat and rice. Our findings indicate that, for these traits in the gene-pool population, a considerable portion of predictive ability is derived from across sub-population genetic variance (i.e. the model’s ability to classify individuals into their respective sub-populations), rather than solely from within sub-population genetic variance (i.e. predictive ability attributable to LD between markers and QTLs). This result is consistent with the observations of Daetwyler et al. (2012), who reported a decline in GEBV accuracy when population structure was accounted for and argued that the reduced accuracy reflects the predictive power attributable to LD between markers and QTLs.

The relatively larger gains in predictive ability with GWAS-preselected variants when population structure was accounted for, compared to models without control for population structure, likely reflect the greater contribution of LD information once the confounding effects of population structure are minimized. A previous study in the Australian mango breeding population found that TC, FBC and fruit blush intensity are strongly associated to population structure (Wilkinson et al., 2022). To avoid spurious associations, separating trait-associated loci from loci associated to ancestry is particularly important in this population. Because population structure was already accounted for during GWAS (through inclusion of PCs as fixed effects), the preselected variants are more likely to tag causative QTLs or be in meaningful LD with them, rather than merely reflecting population stratification. In contrast, WGS data contain many markers that may not be in LD with causative loci but can still contribute to predictive ability by capturing population structure. When population structure is explicitly controlled for in the prediction model, these markers provide little useful genetic signal and may introduce noise, leading to a sharper decline in predictive ability compared to models using trait-informative GWAS-preselected variants.

While our findings demonstrate a marked decline in predictive ability after accounting for population structure using fixed PCs, this sharp reduction may reflect over-correction for population structure arising from double-counting population structure effects (Hong et al., 2025). As argued by Janss et al. (2012), incorporating fixed PCs derived from the same GRM used in the random component of the model can redundantly adjust for population structure, thereby diminishing predictive ability by removing genuine genetic signals alongside confounding effects. Future studies should evaluate methods that address this issue, such as the reparameterized GBLUP model of Janss et al. (2012), which enables natural partitioning of across-subpopulation genetic variance due to population structure and within-subpopulation genetic variance that is of primary interest to breeders. Hong et al. (2025) advocated for accounting for population structure using PCs as random effects to avoid the over-correction that may occur when PCs are fitted as fixed effects in GBLUP models. However, in our study, fitting PCs as fixed effects provided conservative estimates of predictive ability, which are likely more transferable to homogeneous breeding populations or across-population predictions.

## 5 Conclusion

Preselecting SNPs from WGS data based on their estimated effects on target traits enhanced predictive ability in mango, particularly when population structure was accounted for. In contrast, limited improvements were observed when population structure was ignored, likely due to inflated prediction estimates. Integrating GWAS-preselected variants with fixed-effect SNPs yielded superior predictive performance, especially for FBC, across models both accounting for or ignoring population structure. This combined approach outperformed models based solely on WGS data, WGS plus fixed-effect SNPs, or GWAS-preselected variants alone. These findings underscore the value of strategic SNP selection and model refinement using prior biological knowledge to maximize the utility of WGS data in genomic prediction. While our results demonstrate the potential of leveraging GWAS-preselected variants, further validation in larger, more homogenous datasets, particularly those reflecting practical breeding scenarios such as across-population or across-generation predictions is recommended to assess robustness and broader applicability. The sharp decline in predictive ability after accounting for population structure highlights its dominant influence in this mango gene pool, emphasizing the need to account for this factor in genetic analysis to distinguish true LD-driven associations from spurious signals arising from subpopulation differences. The identification of several markers associated with key fruit quality traits and tree vigor provides a valuable resource for future marker-assisted selection and functional genomics research in mango. To ensure their reliability and practical utility in breeding programs, these markers should be further validated under realistic breeding scenarios, such as selection within full-sib families. Overall, this research contributes to the optimization of genomic selection strategies in fruit tree breeding programs, offering a promising pathway to accelerate genetic gain in long-lived species where conventional breeding remains time-consuming and resource-intensive. Once validated in practical breeding populations, the use of GWAS-preselected variants in genomic prediction could enable earlier and more accurate selection, thereby reducing breeding cycle length and accelerating cultivar development in mango.

## Data Availability

All data analyzed in this study was previously published by Wilkinson et al. (2025). The whole genome assemblies and annotations for Irwin, Kensington Pride and M. laurina are submitted to the Genome Warehouse under Bioproject nos. PRJCA020898, PRJCA029779, and PRJCA029972, respectively. Raw sequencing reads have been submitted to NCBI under BioProject nos. PRJNA1148201 (Kensington Pride and M. laurina), PRJNA1034099 (Irwin) and PRJNA1175065 (225 M. indica).
