# Supplementary material for: Increased genomic predictive ability in mango using GWAS-preselected variants and fixed-effect SNPs
Source: Front Plant Sci. 2025 Oct 29;16:1664012. doi: 10.3389/fpls.2025.1664012 (PMC12605043; doi:10.3389/fpls.2025.1664012)
Supplement: Supplementary file 1 [file DataSheet1.docx]

Supplementary Tables and Figures

Supplementary Table 1. List of accessions used as validation population for genomic prediction.

| Accession number | Accession name | Accession type |
| --- | --- | --- |
| 1 | Tommy Atkins | cultivar |
| 2 | 95.041.1201 | Advanced selection |
| 3 | Maha Chanook | cultivar |
| 4 | Irwin | cultivar |
| 5 | R2E2 | cultivar |
| 6 | 94.304.4046 | Advanced selection |
| 7 | Royal Special | cultivar |
| 8 | 95.041.1243 | Advanced selection |
| 9 | 95.041.1233 | Advanced selection |
| 10 | B74 Calypso | cultivar |
| 11 | 94.307.4069 | Advanced selection |
| 12 | Willard | cultivar |
| 13 | Kensington Pride | cultivar |
| 14 | Lombok | cultivar |
| 15 | 95.041.1259 | Advanced selection |
| 16 | 07.027.010 | Advanced selection |
| 17 | 08.041.028 | Advanced selection |
| 18 | Fyhn | cultivar |
| 19 | Honey Gold | cultivar |
| 20 | Glenn | cultivar |
| 21 | Creeper | cultivar |
| 22 | Lippens | cultivar |
| 23 | Keitt | cultivar |
| 24 | Van Dyke | cultivar |
| 25 | Haden | cultivar |
| 26 | Kent | cultivar |
| 27 | Padiri | cultivar |
| 28 | Apple | cultivar |
| 29 | Willard H | cultivar |
| 30 | Nam Doc Mai | cultivar |
| 31 | Edward | cultivar |
| 32 | Sensation | cultivar |
| 33 | 96.307.6173 | Advanced selection |
| 34 | 05.002.044 | Advanced selection |
| 35 | 08.012.081 | Advanced selection |
| 36 | 97.026.7072 | Advanced selection |
| 37 | 95.303.1118 | Advanced selection |
| 38 | 96.026.10035 | Advanced selection |
| 39 | 97.302.3088 | Advanced selection |
| 40 | 95.026.9018 | Advanced selection |
| 41 | Hybrid 10 | cultivar |
| 42 | Hybrid 17 | cultivar |

Supplementary Table 2. Summary statistics for phenotypic traits measured in mango trees. Traits include fruit blush colour (FBC), average fruit weight (AFW), fruit firmness (FF), and trunk circumference at ages 9 (TC_9) AND 12 (tc_12). For each trait, the number of observations (N), mean, standard deviation (SD), and coefficient of variation (CV%) are reported.

| Trait | N | mean | SD | CV% |
| --- | --- | --- | --- | --- |
| FBC | 220 | 1.79 | 1.59 | 88.46 |
| AFW | 222 | 346.61 | 146.79 | 42.35 |
| FF | 221 | 1.03 | 0.29 | 28.31 |
| TC_9 | 200 | 50.40 | 10.63 | 21.1 |
| TC_12 | 199 | 56.06 | 10.80 | 19.27 |

Supplementary Table 3. Heritability estimates in mango using base GBLUP models without accounting for population structure across different marker sets and densities. AIC values are in parentheses.

| **Trait** |  | |  | | | **Marker set** | | | | | | | |
| --- | --- | --- | --- | --- | --- | --- | --- | --- | --- | --- | --- | --- | --- |
|  | **LD_10k** | **LD_20k** | | **LD_80k** | **LD_800k** | | **LD_2mil** | **WGS** | **TOP-BLINK** | **TOP_FarmCPU** | **TOP-MLMM** | **TOP-GLM** |  |
| FBC | 1 (286) | 1.0 (261) | | 1.0 (248) | 0.98 (237) | | 0.98 (236) | 0.98 (236) | 0.97 (210) | 1.0 (220) | 1.0 (145) | 0.95 (161) |  |
| AFW | 1.0 (2359) | 1.0 (2343) | | 1.0 (2330) | 0.98 (2324) | | 0.95 (2327) | 0.95 (2328) | 0.96 (2291) | 1.0 (2285) | 1.0 (2132) | 0.76 (2258) |  |
| FF | 0.23 (-1252) | 0.24 (-1254) | | 0.24 (-1254) | 0.26 (-1249) | | 0.26 (-1248) | 0.26 (-1247) | 0.2 (-1343) | 0.2 (-1343) | 0.21 (-1363) | 0.2 (-1344) |  |
| TC | 0.4 (1861) | 0.38 (1855) | | 0.37 (1849) | 0.34 (1843) | | 0.33 (1842) | 0.33 (1841) | 0.41 (1799) | 0.41 (1799) | 0.42 (1789) | 0.41 (1798) |  |

Supplementary Table 4. Heritability estimates in mango using GBLUP models that accounted for population structure across different marker sets and densities. AIC values are in parenthesis.

| **Trait** |  | | | |  | | | | **Marker set** | | | | | | | | | | | | | | |
| --- | --- | --- | --- | --- | --- | --- | --- | --- | --- | --- | --- | --- | --- | --- | --- | --- | --- | --- | --- | --- | --- | --- | --- |
|  | **LD_10k** | **LD_20k** | | **LD_80k** | | | **LD_800k** | | | **LD_2mil** | | **WGS** | | **TOP-BLINK** | | **TOP_FarmCPU** | | **TOP-MLMM** | | **TOP-GLM** | |  |  |
| FBC | 1.0 (225) | | 1.0 (216) | | | 1.0 (210) | | 0.93 (208) | | | 0.93 (208) | | 0.95 (207) | | 0.92 (179) | | 1.0 (194) | | 0.92 (106) | | 0.76 (123) | |  |
| AFW | 1.0 (2337) | | 1.0 (2330) | | | 1.0 (2323) | | 0.97 (2323) | | | 0.94 (2325) | | 0.93 (2326) | | 1 (2284) | | 1.0 (2298) | | 0.93 (2118) | | 0.78 (2249) | |  |
| FF | 0.27 (-1242) | | 0.26 (-1248) | | | 0.26 (-1249) | | 0.24 (-1254) | | | 0.24 (-1254) | | 0.23 (-1252) | | 0.2 (-1343) | | 0.2 (-1343) | | 0.21 (-1363) | | 0.2 (-1344) | |  |
| TC | 0.39 (1862) | | 0.16 (1994) | | | 0.29 (1905) | | 0.26 (1888) | | | 0.29 (1866) | | 0.32 (1843) | | 0.41 (1797) | | 0.41 (1797) | | 0.42 (1786) | | 0.41 (1795) | |  |

Supplementary Table 5. Predictive abilities for mango traits using GBLUP models without control for population structure under parental validation. Estimates of prediction bias are in parentheses. The density of GWAS-preselected variants (Preselected SNP number) is given in the second column.

| **Trait** | **Preselected SNP number** | **Marker set** | | | | | | | | | |
| --- | --- | --- | --- | --- | --- | --- | --- | --- | --- | --- | --- |
|  |  | **LD_10k** | **LD_20k** | **LD_80k** | **LD_800k** | **LD_2mil** | **WGS** | **TOP-BLINK** | **TOP_FarmCPU** | **TOP-MLMM** | **TOP-GLM** |
| FBC | 1,000 | 0.60 (0.85) | 0.65 (0.85) | 0.65 (0.77) | 0.66 (0.75) | 0.66 (0.75) | 0.67 (0.76) | 0.68 (0.78) | 0.56 (0.67) | 0.64 (0.77) | 0.58 (0.74) |
|  | 10,000 | - | - | - | - | - | - | 0.64 (0.78) | 0.66 (0.77) | 0.66 (0.73) | 0.66 (0.75) |
|  | 15,000 | - | - | - | - | - | - | 0.63 (0.75) | 0.67 (0.74) | 0.67 (0.72) | 0.66 (0.72) |
|  | 20,000 | - | - | - | - | - | - | 0.63 (0.76) | 0.69 (075) | 0.67 (0.73) | 0.66 (0.73) |
|  | 30,000 | - | - | - | - | - | - | 0.66 (0.75) | 0.70 (0.74) | 0.65 (0.7) | 0.65 (0.72) |
|  | 50,000 | - | - | - | - | - | - | 0.68 (0.76) | 0.7 (0.77) | 0.64 (0.7) | 0.65 (0.73) |
|  | 100,000 | - | - | - | - | - | - | 0.7 (0.8) | 0.71 (0.78) | 0.65 (0.72) | 0.66 (0.76) |
| AFW | 1,000 | 0.59 (1.8) | 0.65 (1.77) | 0.66 (1.4) | 0.67 (1.4) | 0.67 (1.46) | 0.67 (1.5) | 0.4 (0.88) | 0.66 (1.31) | 0.7 (1.7) | 0.6 (1.34) |
|  | 10,000 | - | - | - | - | - | - | 0.73 (1.47) | 0.68 (1.32) | 0.7 (1.43) | 0.67 (1.48) |
|  | 15,000 | - | - | - | - | - | - | 0.77 (1.5) | 0.67 (1.25) | 0.69 (1.41) | 0.66 (1.46) |
|  | 20,000 | - | - | - | - | - | - | 0.78 (1.55) | 0.65 (1.22) | 0.69 (1.42) | 0.67 (1.52) |
|  | 30,000 | - | - | - | - | - | - | 0.75 (1.55) | 0.66 (1.24) | 0.69 (1.44) | 0.68 (1.56) |
|  | 50,000 | - | - | - | - | - | - | 0.71 (1.44) | 0.67 (1.28) | 0.68 (1.43) | 0.68 (1.51) |
|  | 100,000 | - | - | - | - | - | - | 0.7 (0.7) | 0.66 (1.32) | 0.67 (1.41) | 0.68 (1.55) |
| FF | 1,000 | 0.35 (1.74) | 0.38 (1.64) | 0.4 (1.56) | 0.41 (1.4) | 0.41 (1.4) | 0.41 (1.39) | 0.43 (0.99) | 0.43 (0.99) | 0.4 (0.95) | 0.45 (1.03) |
|  | 10,000 | - | - | - | - | - | - | 0.37 (0.94) | 0.37 (0.94) | 0.33 (0.82) | 0.38 (0.97) |
|  | 15,000 | - | - | - | - | - | - | 0.38 (0.97) | 0.38 (0.97) | 0.33 (0.82) | 0.38 (0.98) |
|  | 20,000 | - | - | - | - | - | - | 0.37 (0.97) | 0.37 (0.97) | 0.33 (0.82) | 0.38 (0.99) |
|  | 30,000 | - | - | - | - | - | - | 0.36 (0.95) | 0.36 (0.95) | 0.33 (0.84) | 0.37 (0.97) |
|  | 50,000 | - | - | - | - | - | - | 0.37 (0.98) | 0.37 (0.98) | 0.34 (0.87) | 0.37 (0.98) |
|  | 100,000 | - | - | - | - | - | - | 0.37 (1.0) | 0.37 (1.0) | 0.35 (0.9) | 0.37 (0.99) |
| TC | 1,000 | 0.51 (1.24) | 0.52 (1.1) | 0.53 (1.0) | 0.54 (0.93) | 0.54 (0.93) | 0.54 (0.94) | 0.58 (0.85) | 0.58 (0.85) | 0.46 (0.68) | 0.55 (0.86) |
|  | 10,000 | - | - | - | - | - | - | 0.58 (0.79) | 0.58 (0.79) | 0.54 (0.72) | 0.57 (0.8) |
|  | 15,000 | - | - | - | - | - | - | 0.58 (0.78) | 0.58 (0.78) | 0.54 (0.72) | 0.58 (0.8) |
|  | 20,000 | - | - | - | - | - | - | 0.59 (0.77) | 0.59 (0.77) | 0.54 (0.72) | 0.58 (0.8) |
|  | 30,000 | - | - | - | - | - | - | 0.58 (0.77) | 0.58 (0.77) | 0.53 (0.72) | 0.58 (0.78) |
|  | 50,000 | - | - | - | - | - | - | 0.59 (0.77) | 0.59 (0.77) | 0.53 (0.72) | 0.58 (0.76) |
|  | 100,000 | - | - | - | - | - | - | 0.58 (0.77) | 0.58 (0.77) | 0.53 (0.72) | 0.57 (0.74) |

Supplementary Table 6. Predictive abilities for mango traits using GBLUP models while controlling for population structure under parental validation. The density of GWAS-preselected variants (Preselected SNP number) is given in the second column.

| **Trait** | **Preselected SNP number** | **Marker set** | | | | | | | | | |
| --- | --- | --- | --- | --- | --- | --- | --- | --- | --- | --- | --- |
|  |  | **LD_10k** | **LD_20k** | **LD_80k** | **LD_800k** | **LD_2mil** | **WGS** | **TOP-BLINK** | **TOP_FarmCPU** | **TOP-MLMM** | **TOP-GLM** |
| FBC | 1,000 | 0.25 | 0.33 | 0.30 | 0.39 | 0.42 | 0.44 | 0.25 | 0.27 | 0.28 | 0.31 |
|  | 10,000 | - | - | - | - | - | - | 0.28 | 0.35 | 0.33 | 0.33 |
|  | 15,000 | - | - | - | - | - | - | 0.31 | 0.38 | 0.37 | 0.32 |
|  | 20,000 | - | - | - | - | - | - | 0.32 | 0.45 | 0.36 | 0.28 |
|  | 30,000 | - | - | - | - | - | - | 0.41 | 0.44 | 0.33 | 0.26 |
|  | 50,000 | - | - | - | - | - | - | 0.45 | 0.50 | 0.27 | 0.23 |
|  | 100,000 | - | - | - | - | - | - | 0.42 | 0.50 | 0.26 | 0.21 |
| AFW | 1,000 | 0.33 | 0.40 | 0.45 | 0.37 | 0.34 | 0.30 | 0.10 | 0.44 | 0.39 | 0.54 |
|  | 10,000 | - | - | - | - | - | - | 0.30 | 0.57 | 0.46 | 0.53 |
|  | 15,000 | - | - | - | - | - | - | 0.31 | 0.58 | 0.47 | 0.52 |
|  | 20,000 | - | - | - | - | - | - | 0.29 | 0.57 | 0.48 | 0.52 |
|  | 30,000 | - | - | - | - | - | - | 0.29 | 0.54 | 0.47 | 0.52 |
|  | 50,000 | - | - | - | - | - | - | 0.33 | 0.50 | 0.46 | 0.51 |
|  | 100,000 | - | - | - | - | - | - | 0.37 | 0.47 | 0.44 | 0.51 |
| FF | 1,000 | 0.16 | 0.19 | 0.26 | 0.28 | 0.29 | 0.30 | 0.34 | 0.34 | 0.27 | 0.35 |
|  | 10,000 | - | - | - | - | - | - | 0.22 | 0.22 | 0.21 | 0.22 |
|  | 15,000 | - | - | - | - | - | - | 0.22 | 0.22 | 0.20 | 0.21 |
|  | 20,000 | - | - | - | - | - | - | 0.21 | 0.21 | 0.20 | 0.21 |
|  | 30,000 | - | - | - | - | - | - | 0.19 | 0.19 | 0.20 | 0.19 |
|  | 50,000 | - | - | - | - | - | - | 0.18 | 0.18 | 0.20 | 0.17 |
|  | 100,000 | - | - | - | - | - | - | 0.18 | 0.18 | 0.20 | 0.16 |
| TC | 1,000 | 0.55 | 0.57 | 0.58 | 0.58 | 0.58 | 0.57 | 0.58 | 0.58 | 0.48 | 0.55 |
|  | 10,000 | - | - | - | - | - | - | 0.60 | 0.60 | 0.55 | 0.58 |
|  | 15,000 | - | - | - | - | - | - | 0.60 | 0.60 | 0.55 | 0.59 |
|  | 20,000 | - | - | - | - | - | - | 0.60 | 0.60 | 0.55 | 0.58 |
|  | 30,000 | - | - | - | - | - | - | 0.61 | 0.61 | 0.55 | 0.59 |
|  | 50,000 | - | - | - | - | - | - | 0.61 | 0.61 | 0.55 | 0.60 |
|  | 100,000 | - | - | - | - | - | - | 0.60 | 0.60 | 0.55 | 0.59 |

Supplementary Table 7. Predictive abilities for mango traits under 5-fold cross validation using GBLUP models without accounting for population structure. The density of GWAS-preselected variants (Preselected SNP number) is given in the second column.

| **Trait** | **Preselected SNP number** | **Marker set** | | | | | | | | | |
| --- | --- | --- | --- | --- | --- | --- | --- | --- | --- | --- | --- |
|  |  | **LD_10k** | **LD_20k** | **LD_80k** | **LD_800k** | **LD_2mil** | **WGS** | **TOP-BLINK** | **TOP_FarmCPU** | **TOP-MLMM** | **TOP-GLM** |
| FBC | 1,000 | 0.78 | 0.79 | 0.79 | 0.80 | 0.80 | 0.80 | 0.73 | 0.74 | 0.73 | 0.76 |
|  | 10,000 | - | - | - | - | - | - | 0.75 | 0.78 | 0.77 | 0.80 |
|  | 15,000 | - | - | - | - | - | - | 0.76 | 0.78 | 0.77 | 0.80 |
|  | 20,000 | - | - | - | - | - | - | 0.76 | 0.78 | 0.77 | 0.80 |
|  | 30,000 | - | - | - | - | - | - | 0.77 | 0.79 | 0.78 | 0.80 |
|  | 50,000 | - | - | - | - | - | - | 0.78 | 0.79 | 0.79 | 0.81 |
|  | 100,000 | - | - | - | - | - | - | 0.78 | 0.80 | 0.79 | 0.81 |
| AFW | 1,000 | 0.64 | 0.66 | 0.66 | 0.65 | 0.65 | 0.65 | 0.57 | 0.58 | 0.63 | 0.61 |
|  | 10,000 | - | - | - | - | - | - | 0.63 | 0.60 | 0.64 | 0.66 |
|  | 15,000 | - | - | - | - | - | - | 0.63 | 0.61 | 0.64 | 0.66 |
|  | 20,000 | - | - | - | - | - | - | 0.63 | 0.62 | 0.64 | 0.66 |
|  | 30,000 | - | - | - | - | - | - | 0.63 | 0.62 | 0.65 | 0.66 |
|  | 50,000 | - | - | - | - | - | - | 0.63 | 0.63 | 0.65 | 0.66 |
|  | 100,000 | - | - | - | - | - | - | 0.64 | 0.64 | 0.65 | 0.66 |
| FF | 1,000 | 0.31 | 0.33 | 0.31 | 0.29 | 0.28 | 0.28 | 0.27 | 0.28 | 0.29 | 0.28 |
|  | 10,000 | - | - | - | - | - | - | 0.27 | 0.27 | 0.27 | 0.27 |
|  | 15,000 | - | - | - | - | - | - | 0.27 | 0.27 | 0.27 | 0.27 |
|  | 20,000 | - | - | - | - | - | - | 0.27 | 0.27 | 0.27 | 0.27 |
|  | 30,000 | - | - | - | - | - | - | 0.28 | 0.28 | 0.28 | 0.28 |
|  | 50,000 | - | - | - | - | - | - | 0.27 | 0.28 | 0.28 | 0.28 |
|  | 100,000 | - | - | - | - | - | - | 0.28 | 0.28 | 0.28 | 0.28 |
| TC | 1,000 | 0.56 | 0.56 | 0.57 | 0.57 | 0.57 | 0.57 | 0.52 | 0.51 | 0.52 | 0.53 |
|  | 10,000 | - | - | - | - | - | - | 0.54 | 0.55 | 0.54 | 0.55 |
|  | 15,000 | - | - | - | - | - | - | 0.55 | 0.56 | 0.54 | 0.56 |
|  | 20,000 | - | - | - | - | - | - | 0.55 | 0.56 | 0.55 | 0.56 |
|  | 30,000 | - | - | - | - | - | - | 0.55 | 0.56 | 0.55 | 0.56 |
|  | 50,000 | - | - | - | - | - | - | 0.56 | 0.56 | 0.56 | 0.56 |
|  | 100,000 | - | - | - | - | - | - | 0.56 | 0.56 | 0.56 | 0.56 |

Supplementary Table 8. Predictive abilities for mango traits under 5-fold cross validation using GBLUP models while controlling for population structure. The density of GWAS-preselected variants (Preselected SNP number) is given in the second column.

| **Trait** | **Preselected SNP number** | **Marker set** | | | | | | | | | |
| --- | --- | --- | --- | --- | --- | --- | --- | --- | --- | --- | --- |
|  |  | **LD_10k** | **LD_20k** | **LD_80k** | **LD_800k** | **LD_2mil** | **WGS** | **TOP-BLINK** | **TOP_FarmCPU** | **TOP-MLMM** | **TOP-GLM** |
| FBC | 1,000 | 0.31 | 0.38 | 0.32 | 0.38 | 0.38 | 0.35 | 0.26 | 0.20 | 0.25 | 0.40 |
|  | 10,000 | - | - | - | - | - | - | 0.24 | 0.30 | 0.26 | 0.45 |
|  | 15,000 | - | - | - | - | - | - | 0.27 | 0.30 | 0.26 | 0.44 |
|  | 20,000 | - | - | - | - | - | - | 0.28 | 0.31 | 0.27 | 0.44 |
|  | 30,000 | - | - | - | - | - | - | 0.29 | 0.33 | 0.29 | 0.43 |
|  | 50,000 | - | - | - | - | - | - | 0.30 | 0.36 | 0.31 | 0.42 |
|  | 100,000 | - | - | - | - | - | - | 0.34 | 0.39 | 0.33 | 0.40 |
| AFW | 1,000 | 0.35 | 0.38 | 0.41 | 0.35 | 0.34 | 0.32 | 0.33 | 0.39 | 0.48 | 0.41 |
|  | 10,000 | - | - | - | - | - | - | 0.36 | 0.37 | 0.47 | 0.43 |
|  | 15,000 | - | - | - | - | - | - | 0.36 | 0.38 | 0.47 | 0.43 |
|  | 20,000 | - | - | - | - | - | - | 0.36 | 0.39 | 0.47 | 0.44 |
|  | 30,000 | - | - | - | - | - | - | 0.36 | 0.40 | 0.47 | 0.44 |
|  | 50,000 | - | - | - | - | - | - | 0.36 | 0.39 | 0.47 | 0.43 |
|  | 100,000 | - | - | - | - | - | - | 0.36 | 0.40 | 0.47 | 0.43 |
| FF | 1,000 | 0.19 | 0.21 | 0.19 | 0.12 | 0.10 | 0.08 | 0.20 | 0.20 | 0.20 | 0.20 |
|  | 10,000 | - | - | - | - | - | - | 0.17 | 0.17 | 0.18 | 0.17 |
|  | 15,000 | - | - | - | - | - | - | 0.17 | 0.17 | 0.18 | 0.17 |
|  | 20,000 | - | - | - | - | - | - | 0.17 | 0.17 | 0.18 | 0.18 |
|  | 30,000 | - | - | - | - | - | - | 0.16 | 0.17 | 0.18 | 0.17 |
|  | 50,000 | - | - | - | - | - | - | 0.16 | 0.16 | 0.18 | 0.16 |
|  | 100,000 | - | - | - | - | - | - | 0.15 | 0.16 | 0.16 | 0.16 |
| TC | 1,000 | 0.53 | 0.50 | 0.47 | 0.48 | 0.47 | 0.45 | 0.46 | 0.48 | 0.46 | 0.48 |
|  | 10,000 | - | - | - | - | - | - | 0.49 | 0.50 | 0.47 | 0.50 |
|  | 15,000 | - | - | - | - | - | - | 0.49 | 0.50 | 0.48 | 0.50 |
|  | 20,000 | - | - | - | - | - | - | 0.49 | 0.50 | 0.48 | 0.50 |
|  | 30,000 | - | - | - | - | - | - | 0.49 | 0.50 | 0.49 | 0.50 |
|  | 50,000 | - | - | - | - | - | - | 0.49 | 0.50 | 0.49 | 0.50 |
|  | 100,000 | - | - | - | - | - | - | 0.49 | 0.50 | 0.49 | 0.50 |

Supplementary Table 9. Predictive abilities of mango traits using GBLUP models without control for population structure + fixed-effect SNPs under parental validation. The density of GWAS-preselected variants (Preselected SNP number) is given in the second column.

| **Trait** | **Preselected SNP number** | **Marker set** | | | | | | | | | |
| --- | --- | --- | --- | --- | --- | --- | --- | --- | --- | --- | --- |
|  |  | **LD_10k** | **LD_20k** | **LD_80k** | **LD_800k** | **LD_2mil** | **WGS** | **TOP-BLINK** | **TOP_FarmCPU** | **TOP-MLMM** | **TOP-GLM** |
| FBC | 1,000 | 0.73 | 0.75 | 0.74 | 0.73 | 0.73 | 0.74 | 0.74 | 0.68 | 0.65 | 0.60 |
|  | 10,000 | - | - | - | - | - | - | 0.74 | 0.73 | 0.67 | 0.67 |
|  | 15,000 | - | - | - | - | - | - | 0.73 | 0.72 | 0.68 | 0.67 |
|  | 20,000 | - | - | - | - | - | - | 0.73 | 0.72 | 0.68 | 0.68 |
|  | 30,000 | - | - | - | - | - | - | 0.74 | 0.73 | 0.67 | 0.67 |
|  | 50,000 | - | - | - | - | - | - | 0.76 | 0.72 | 0.67 | 0.68 |
|  | 100,000 | - | - | - | - | - | - | 0.77 | 0.74 | 0.68 | 0.70 |
| AFW | 1,000 | 0.60 | 0.65 | 0.67 | 0.67 | 0.67 | 0.68 | 0.41 | 0.69 | 0.71 | 0.60 |
|  | 10,000 | - | - | - | - | - | - | 0.73 | 0.69 | 0.70 | 0.68 |
|  | 15,000 | - | - | - | - | - | - | 0.78 | 0.68 | 0.69 | 0.67 |
|  | 20,000 | - | - | - | - | - | - | 0.78 | 0.66 | 0.69 | 0.68 |
|  | 30,000 | - | - | - | - | - | - | 0.76 | 0.67 | 0.69 | 0.69 |
|  | 50,000 | - | - | - | - | - | - | 0.72 | 0.67 | 0.68 | 0.69 |
|  | 100,000 | - | - | - | - | - | - | 0.70 | 0.67 | 0.67 | 0.69 |
| TC | 1,000 | 0.52 | 0.54 | 0.56 | 0.58 | 0.58 | 0.58 | 0.63 | 0.63 | 0.54 | 0.60 |
|  | 10,000 | - | - | - | - | - | - | 0.64 | 0.64 | 0.61 | 0.62 |
|  | 15,000 | - | - | - | - | - | - | 0.64 | 0.64 | 0.61 | 0.63 |
|  | 20,000 | - | - | - | - | - | - | 0.64 | 0.64 | 0.61 | 0.63 |
|  | 30,000 | - | - | - | - | - | - | **0.64** | **0.64** | 0.51 | 0.63 |
|  | 50,000 | - | - | - | - | - | - | **0.64** | **0.64** | 0.60 | 0.64 |
|  | 100,000 | - | - | - | - | - | - | 0.64 | 0.64 | 0.60 | 0.63 |

Supplementary Table 10. Predictive abilities of mango traits using GBLUP models with control for population structure + fixed-effect SNPs under parental validation. The density of GWAS-preselected variants (Preselected SNP number) is given in the second column.

| **Trait** | **Preselected SNP number** | **Marker set** | | | | | | | | | |
| --- | --- | --- | --- | --- | --- | --- | --- | --- | --- | --- | --- |
|  |  | **LD_10k** | **LD_20k** | **LD_80k** | **LD_800k** | **LD_2mil** | **WGS** | **TOP-BLINK** | **TOP_FarmCPU** | **TOP-MLMM** | **TOP-GLM** |
| FBC | 1,000 | 0.59 | 0.61 | 0.57 | 0.60 | 0.60 | 0.62 | 0.57 | 0.60 | 0.43 | 0.43 |
|  | 10,000 | - | - | - | - | - | - | 0.62 | 0.64 | 0.48 | 0.48 |
|  | 15,000 | - | - | - | - | - | - | 0.63 | 0.63 | 0.51 | 0.48 |
|  | 20,000 | - | - | - | - | - | - | 0.63 | 0.64 | 0.51 | 0.48 |
|  | 30,000 | - | - | - | - | - | - | 0.66 | 0.62 | 0.50 | 0.47 |
|  | 50,000 | - | - | - | - | - | - | 0.69 | 0.62 | 0.48 | 0.47 |
|  | 100,000 | - | - | - | - | - | - | 0.68 | 0.62 | 0.49 | 0.47 |
| AFW | 1,000 | 0.34 | 0.40 | 0.45 | 0.38 | 0.34 | 0.30 | 0.11 | 0.44 | 0.39 | 0.55 |
|  | 10,000 | - | - | - | - | - | - | 0.30 | 0.57 | 0.46 | 0.53 |
|  | 15,000 | - | - | - | - | - | - | 0.31 | 0.59 | 0.48 | 0.52 |
|  | 20,000 | - | - | - | - | - | - | 0.29 | 0.58 | 0.48 | 0.53 |
|  | 30,000 | - | - | - | - | - | - | 0.29 | 0.55 | 0.48 | 0.52 |
|  | 50,000 | - | - | - | - | - | - | 0.33 | 0.50 | 0.47 | 0.52 |
|  | 100,000 | - | - | - | - | - | - | 0.36 | 0.47 | 0.44 | 0.52 |
| TC | 1,000 | 0.55 | 0.58 | 0.59 | 0.61 | 0.62 | 0.61 | 0.63 | 0.63 | 0.55 | 0.60 |
|  | 10,000 | - | - | - | - | - | - | 0.65 | 0.65 | 0.61 | 0.62 |
|  | 15,000 | - | - | - | - | - | - | 0.65 | 0.65 | 0.62 | 0.64 |
|  | 20,000 | - | - | - | - | - | - | 0.65 | 0.65 | 0.61 | 0.64 |
|  | 30,000 | - | - | - | - | - | - | 0.65 | 0.65 | 0.61 | 0.64 |
|  | 50,000 | - | - | - | - | - | - | 0.66 | 0.66 | 0.61 | 0.65 |
|  | 100,000 | - | - | - | - | - | - | 0.65 | 0.65 | 0.61 | 0.65 |


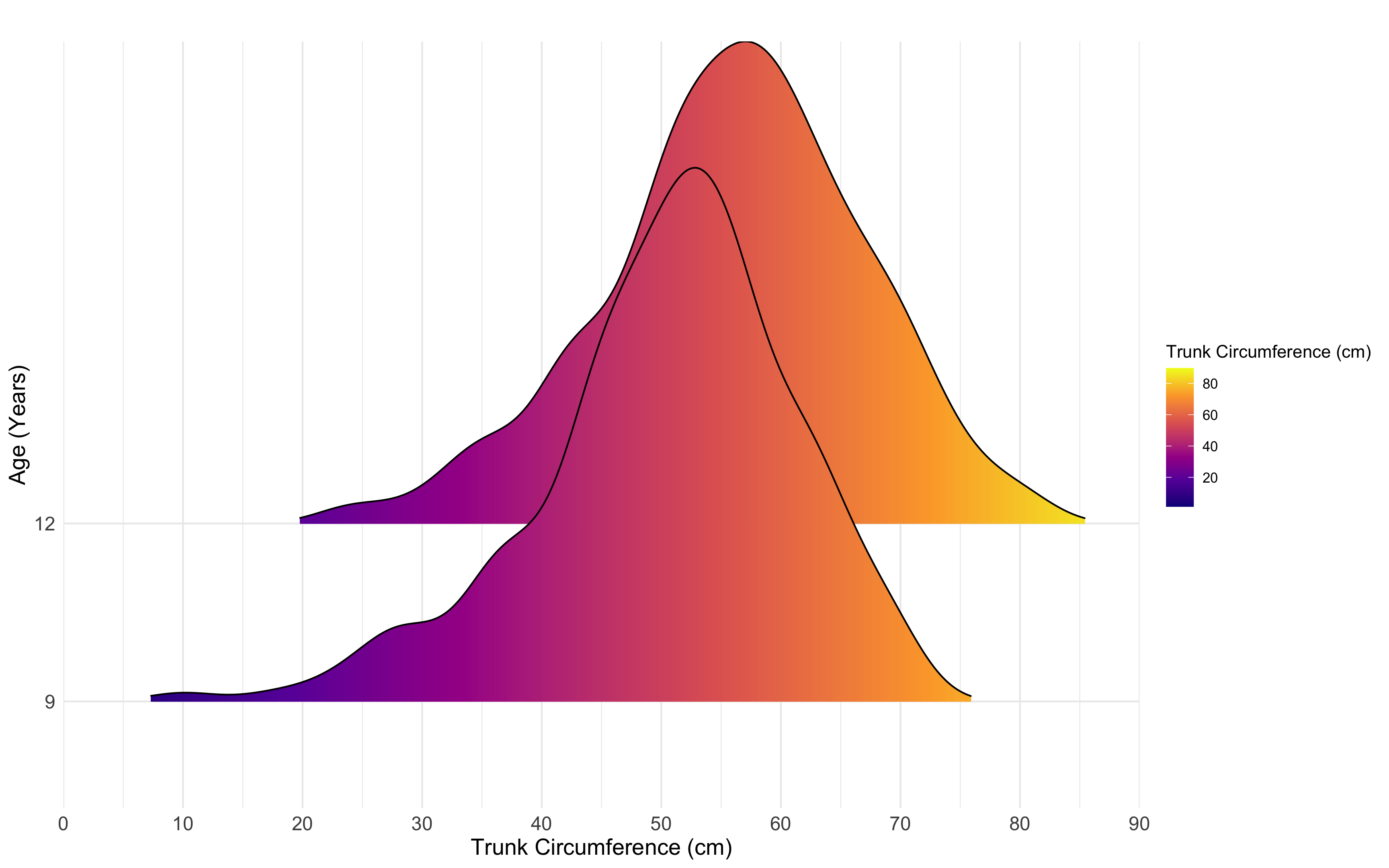


Supplementary Figure 1. Density distribution of trunk circumference measurements in mango trees assessed at ages 9 and 12 years. The color gradient from purple to yellow represents increasing trunk circumference. The plot highlights age-related growth trends and phenotypic variation.


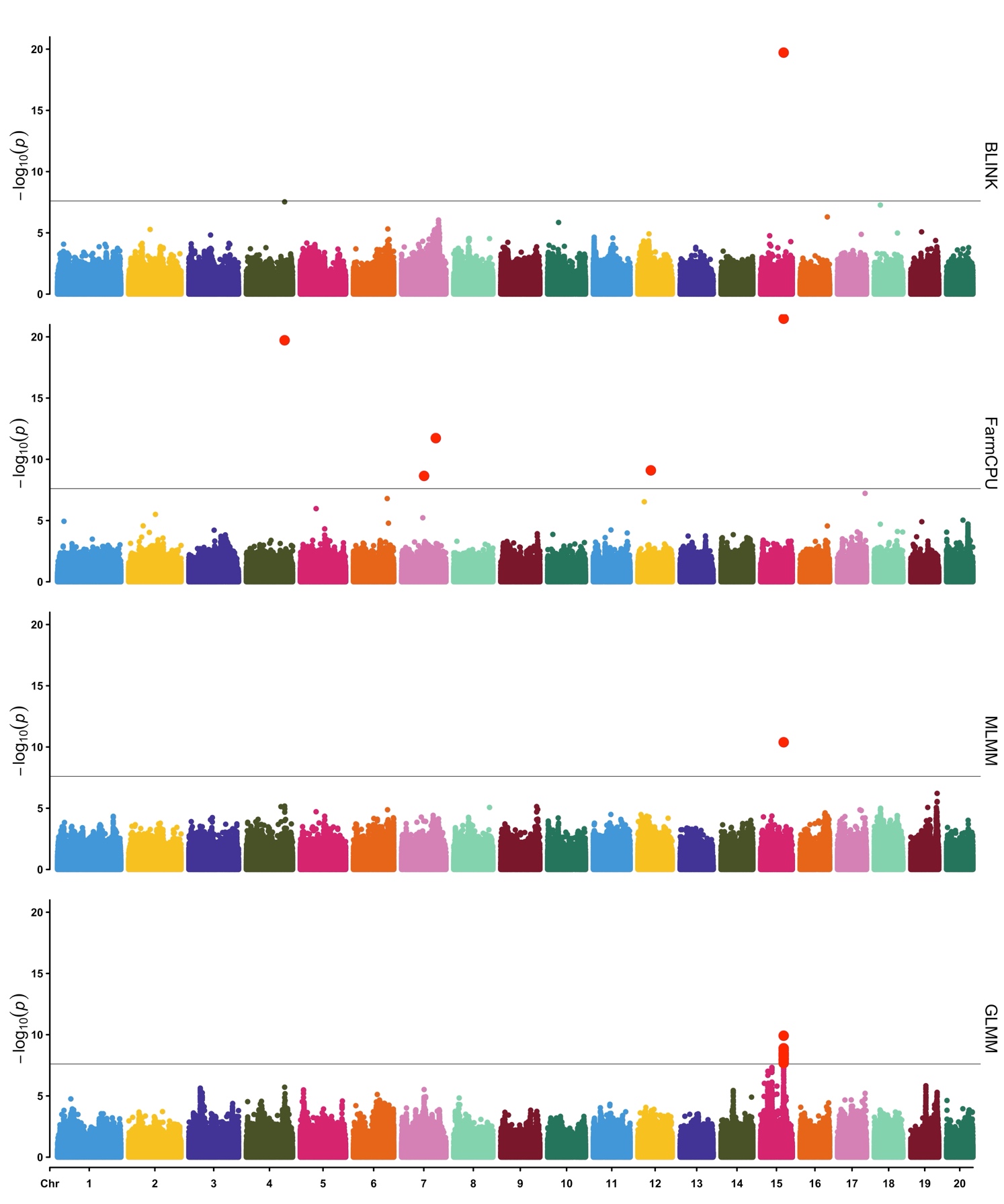


Supplementary Figure 2. GWAS Manhattan plot for fruit blush color in mango. Results based on ~2 million SNPs, analyzed using three multi-locus GWAS (BLINK, FarmCPU, MLMM) and one single-locus GWAS (GLMM). Significant SNPs are highlighted at the Bonferroni threshold (-log(p) = 7.61). The GWAS method is shown on far-right side of the Manhattan plot.


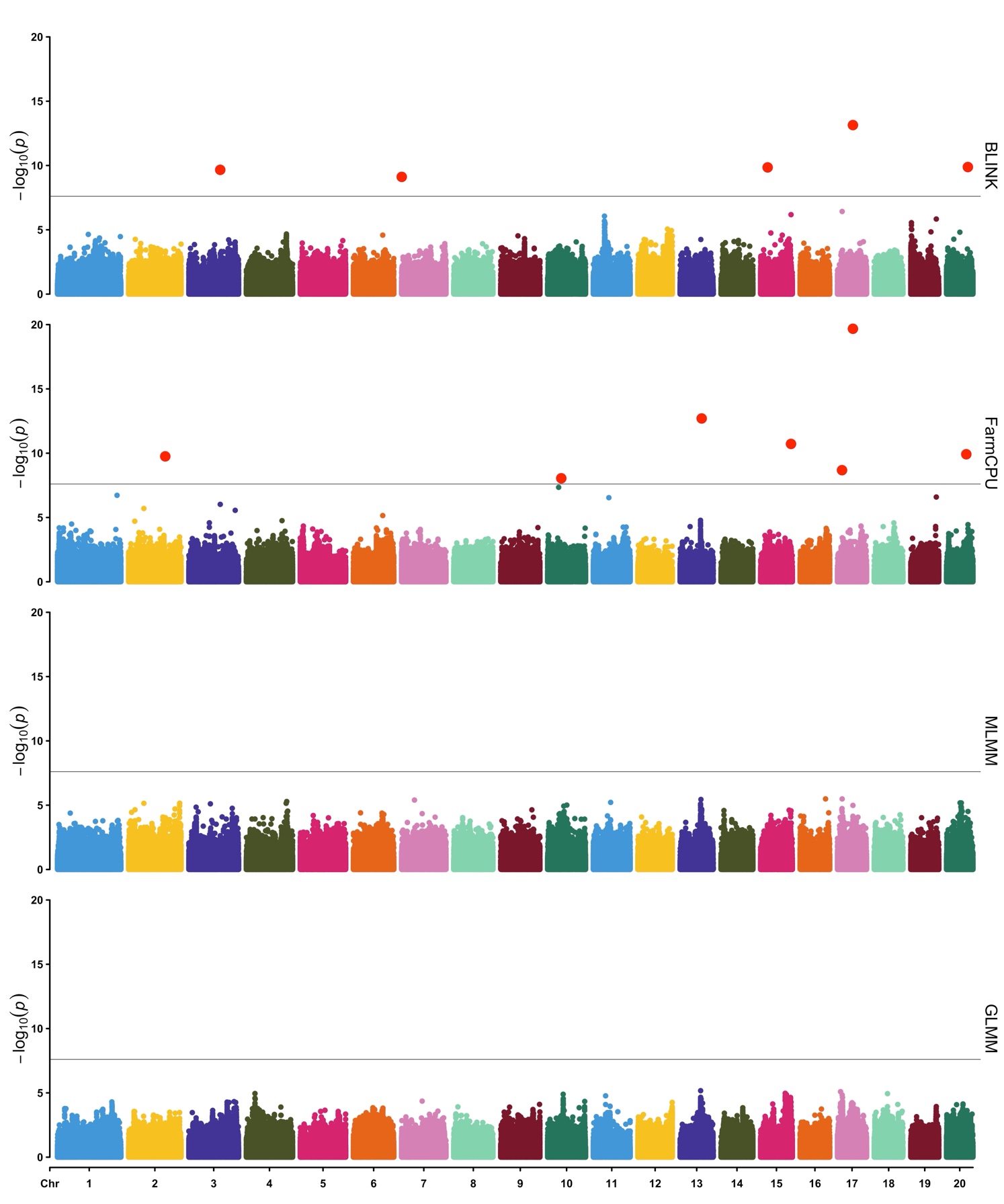


Supplementary Figure 3. Manhattan plot for fruit weight in mango. Results based on ~2 million SNPs analyzed using three multi-locus GWAS (BLINK, FarmCPU, MLMM) and one single-locus GWAS (GLMM). Significant SNPs are highlighted at the Bonferroni threshold (-log(p) = 7.61). The GWAS method is shown on far-right side of the Manhattan plot.


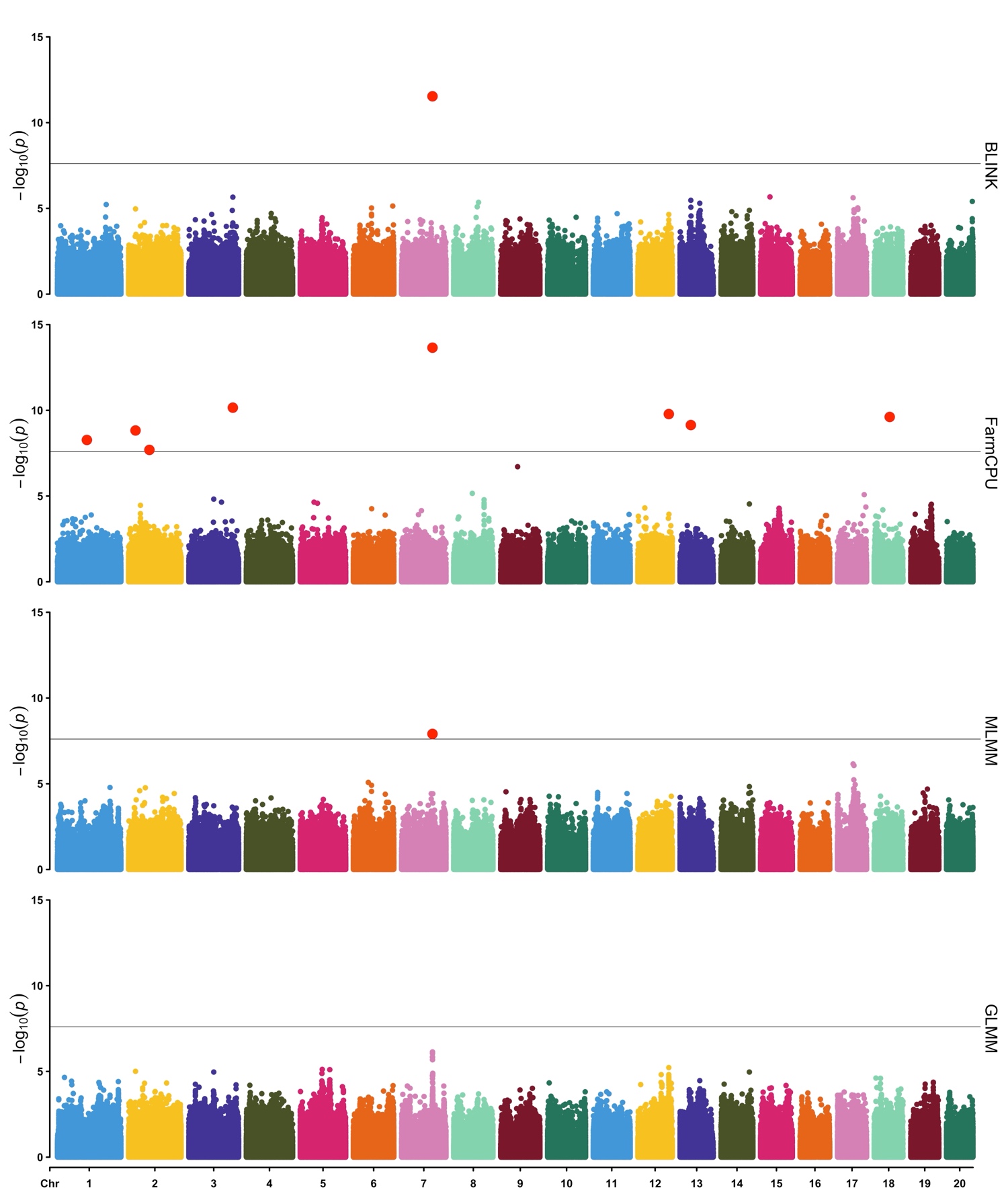


Supplementary Figure 4. GWAS Manhattan plot for trunk circumference. Results based on ~2 million SNPs analyzed using three multi-locus GWAS (BLINK, FarmCPU, MLMM) and one single-locus GWAS (GLMM). Significant SNPs are shown at the Bonferroni threshold (-log(p) = 7.61). The GWAS method is shown on far-right side of the Manhattan plot.


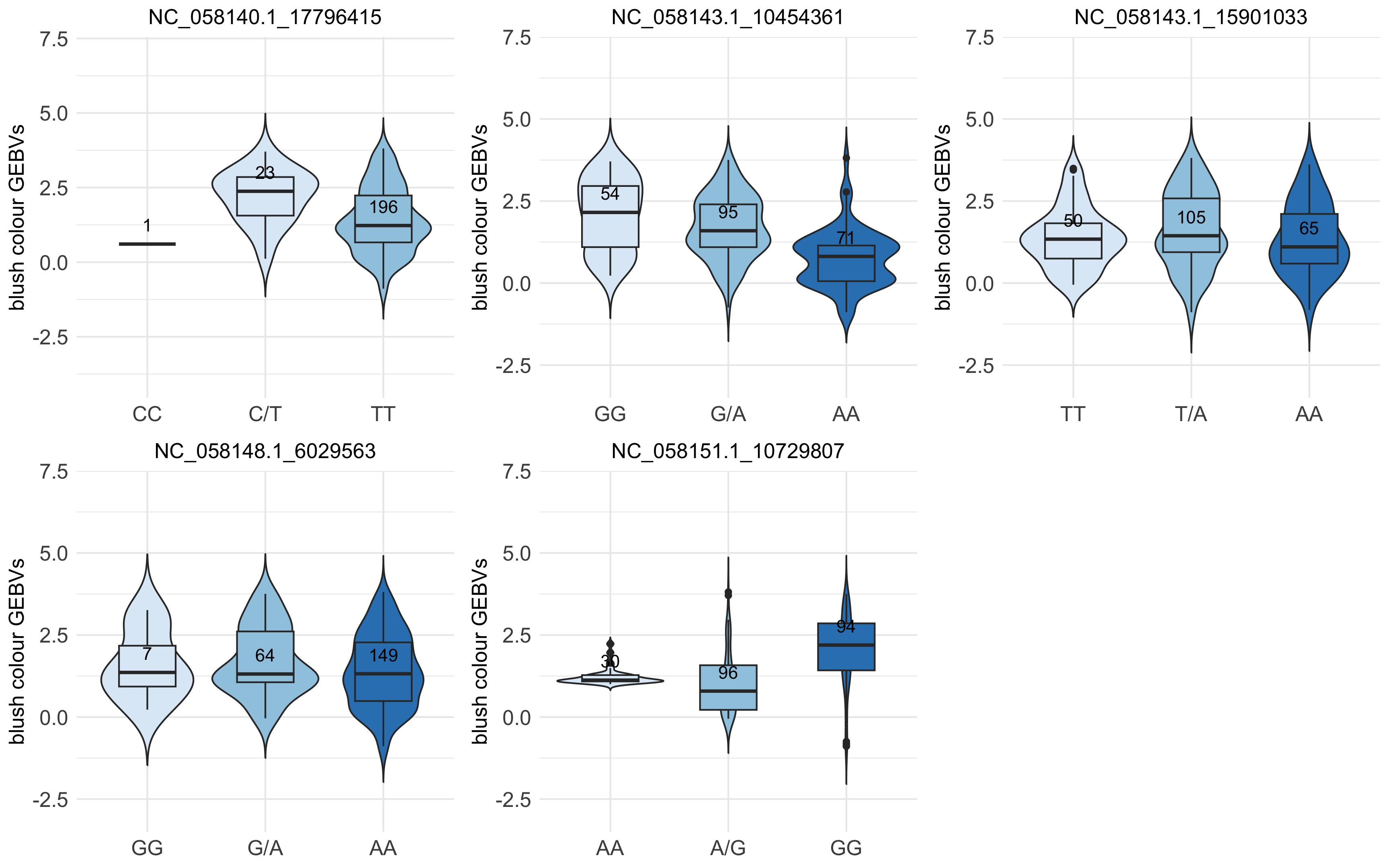


Supplementary Figure 5. Violin plots showing the frequency distribution of fruit blush color GEBVs across different genotypes. The X-axis in each violin plot represent the genotypes, with the first allele (the one on the left) depicting the homozygous genotype for the reference allele, the second (middle) allele indicating the heterozygous genotype, and the third (on the right) representing the homozygous genotype for the minor or alternative allele. The name of the SNP is shown at the top of each violin plot. The reliable SNP (i.e., identified by at least two GWAS methods) for fruit blush color is NC_058151.1_10729807.


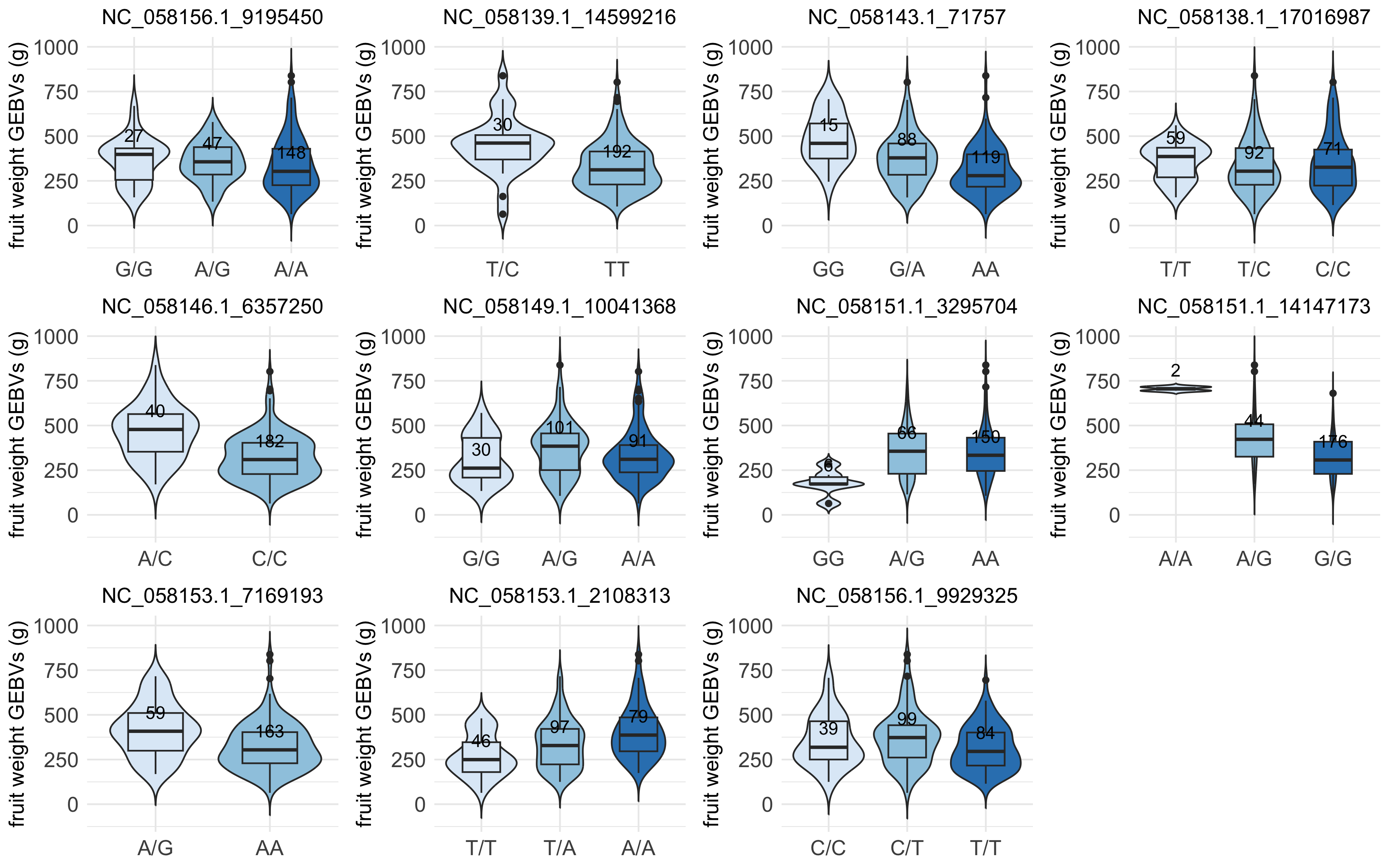


Supplementary Figure 6. Violin plots showing the frequency distribution of average fruit weight GEBVs across different genotypes. The X-axis in each violin plot represent the genotypes, with the first allele (the one on the left) depicting the homozygous genotype for the reference allele, the second (middle) allele indicating the heterozygous genotype, and the third (on the right) representing the homozygous genotype for the minor or alternative allele. The reliable SNP (i.e., identified by at least two GWAS methods) for average fruit weight is NC_058153.1_7169193.


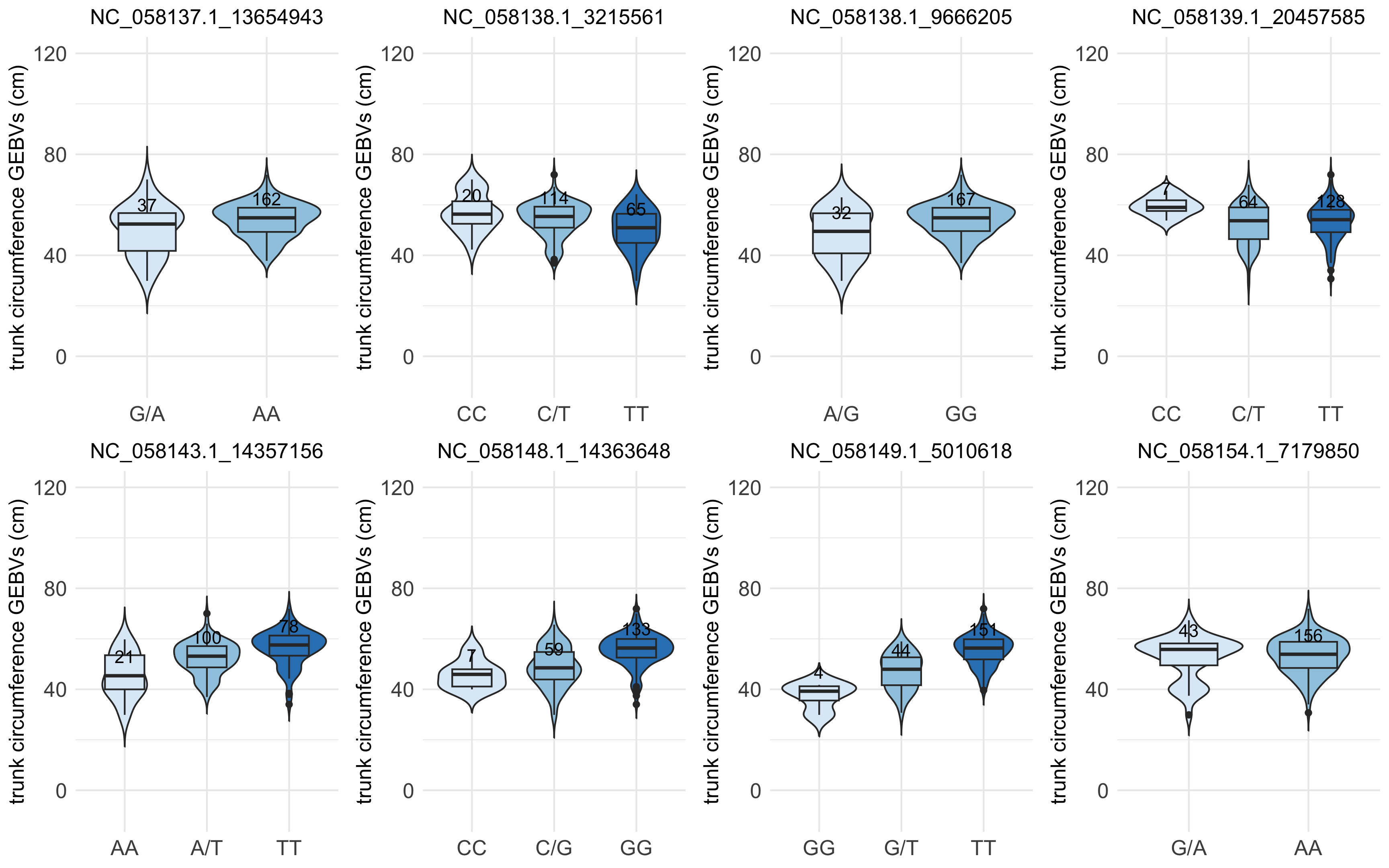


Supplementary Figure 7. Violin plots showing the frequency distribution of trunk circumference GEBVs across different genotypes. The X-axis in each violin plot represent the genotypes, with the first allele (the one on the left) depicting the homozygous genotype for the reference allele, the second (middle) allele indicating the heterozygous genotype, and the third (on the right) representing the homozygous genotype for the minor or alternative allele. The name of the SNP is shown at the top of each violin plot. The reliable SNP (i.e., identified by at least two GWAS methods) for trunk circumference is NC_058143.1_14357156.
